# Gray Matter Microstructure Measured Using Diffusion Imaging as a Biomarker of Severity in Lewy Body Diseases

**DOI:** 10.1002/mds.70355

**Published:** 2026-05-25

**Authors:** Angeliki Zarkali, Ivelina Dobreva, Naomi Hannaway, Rohan Bhome, George E.C. Thomas, Steffani Carrera, Amanda J. Heslegrave, Elena Veleva, Henrik Zetterberg, Rimona S. Weil

**Affiliations:** ^1^ Dementia Research Centre, UCL Queen Square Institute of Neurology University College London London United Kingdom; ^2^ National Hospital for Neurology and Neurosurgery London United Kingdom; ^3^ Movement Disorders Centre, UCL Queen Square Institute of Neurology University College London London United Kingdom; ^4^ Department of Imaging Neuroscience, UCL Queen Square Institute of Neurology University College London London United Kingdom; ^5^ UK DRI Fluid Biomarker Lab and Biomarker Factory University College London London United Kingdom

**Keywords:** diffusion‐weighted imaging, gray matter microstructure, Lewy body dementia, Lewy body diseases, neuroimaging, Parkinson's disease

## Abstract

**Background:**

Despite widespread cortical involvement in Lewy body diseases, conventional gray matter magnetic resonance imaging (MRI) shows limited sensitivity. Diffusion‐weighted MRI‐derived microstructural measures have shown utility in Alzheimer's disease, but their application across the Lewy body disease spectrum remains limited.

**Objectives:**

The goal was to examine gray matter microstructure in 197 participants across the Lewy body disease spectrum (88 Lewy body dementia [LBD], 109 Parkinson's disease, and normal cognition [PD‐NC]) and 46 controls.

**Methods:**

Tissue‐weighted neurite density index, tissue‐weighted orientation dispersion index, and free water fraction were examined across 200 cortical and 32 subcortical regions. We compared these cross‐sectionally between LBD, PD‐NC and controls, and longitudinally over 3 years in a subset of 100 patients with Parkinson's disease cognitively intact at baseline. We assess associations with disease severity and plasma p‐tau217 levels.

**Results:**

LBD showed widespread cortical and subcortical increases in free water fraction and, to lesser extent, orientation dispersion compared to controls and PD‐NC, despite minimal atrophy and no neurite density differences. In temporoparietal regions, increases in free water fraction and orientation dispersion correlated with cognitive, but not motor, severity, and with plasma p‐tau217. Longitudinally, Parkinson's disease patients who developed cognitive impairment showed greater free water fraction increases within bilateral temporal regions, right thalamus, and right caudate.

**Conclusions:**

Diffusion‐derived microstructural measures, particularly free water fraction are sensitive to early and progressive gray matter changes associated with cognitive decline in Lewy body diseases. Findings suggest microstructural disruption without overt neuronal loss and support free water fraction as a potential biomarker for disease staging and progression. © 2026 The Author(s). *Movement Disorders* published by Wiley Periodicals LLC on behalf of International Parkinson and Movement Disorder Society.

Lewy body diseases, including Parkinson's disease (PD), Parkinson's disease dementia (PDD), and dementia with Lewy bodies (DLB) share overlapping clinical features and are characterized by pathological accumulation of α‐synuclein forming Lewy bodies and neurites within cortical and subcortical regions. Despite considerable inter‐individual heterogeneity in α‐synuclein distribution within the brain,^1^, ^2^, ^3^ current neuropathological diagnostic criteria rely on identification of Lewy pathology in gray matter,^4^ and there is evidence of widespread molecular,^5^, ^6^ mitochondrial^7^ and functional neuronal dysfunction.^8^ Additionally, β‐amyloid and tau co‐pathology is frequently found at postmortem and relates to cognitive severity.^9^

Conventional magnetic resonance imaging (MRI) measures of gray matter integrity, such as atrophy, show heterogeneous findings in Lewy body disease.^10^, ^11^, ^12^, ^13^, ^14^ Although higher cortical atrophy rates are associated with worse cognition,^12^, ^15^, ^16^ atrophy is less reliable at earlier stages^14^ and in cognitively intact PD patients.^17^ This suggests that atrophy measures alone are insensitive to early pathology, limiting their utility as biomarkers for early cortical involvement.

Advances in diffusion‐weighted imaging (DWI) allow examination of gray matter microstructure in vivo. Neurite orientation dispersion and density imaging (NODDI) is a biophysical model that separates signal from intra‐neurite, extra‐neurite, and free‐water compartments.^18^ Although mostly applied to white matter, NODDI is particularly well‐suited for gray matter modelling, which mostly comprises of dendrites and crossing axons rather than long‐range fiber tracts. Traditional diffusion metrics are insensitive or ambiguous within such regions of varied orientations.^18^, ^19^, ^20^ NODDI characterizes three microstructural properties: (1) free‐water fraction (FWF), which estimates the volume fraction of free water, contributed primarily from cerebrospinal fluid (CSF), with higher values representing increased extracellular component; (2) orientation dispersion index (ODI) reflecting the spatial configuration of neurites, with higher values reflecting more complex dendritic organization;^18^, ^20^ and (3) neurite density index (NDI), reflecting the volume fraction of neurites, with higher values representing higher neurite density.

Such microstructural metrics show increased sensitivity in capturing gray matter changes associated with healthy aging,^21^, ^22^, ^23^ and Alzheimer's disease pathology^22^, ^24^, ^25^, ^26^, ^27^ even in pre‐symptomatic stages.^21^, ^27^, ^28^ In Lewy body diseases, few studies have assessed gray matter microstructure. Small studies examining changes in PD compared to controls have consistently shown increased FWF in basal ganglia and widespread cortical regions,^17^, ^29^, ^30^ and to lesser extent and less consistently reduced NDI and ODI.^31^, ^32^ In DLB, widespread changes are seen in cortical gray matter compared to controls, with microstructural alterations correlated with β‐amyloid and tau‐positron emission tomography (PET) burden.^33^ However, PD and DLB have mostly been investigated separately with different methodologies and across different disease subgroups (Table 1).

**TABLE 1:** . *Existing studies of gray matter microstructural integrity in Lewy body diseases measured using diffusion‐weighted imaging*

Study	Groups	Methods	Gray‐matter regions analyzed	Main findings	Clinical correlations
PD					
Kamagata et al.^17^	30 PD 28 HC	Two shells (*b* = 1000, *b* = 2000) 64 directions Cross‐sectional GBSS	Harvard–Oxford cortical structural atlas 96 cortical and 17 subcortical regions	↑ FWF, ↓ NDI in caudate and putamen and widespread cortex particularly frontal and temporal regions No differences in ODI between PD and HC No cortical thickness differences	↓ NDI/ODI in frontal, temporal, limbic, basal ganglia and paralimbic regions correlated with UPDRS‐III motor scores ↑ FWF basal ganglia correlated with UPDRS‐III motor scores
Bai et al^30^	38 PD‐MCI 38 PD‐NC 32 HC	Two‐shells (*b* = 1000, *b* = 2000) Cross‐sectional GBSS	Whole cortex	PD‐MCI vs. HC: ↓ ODI in posterior cingulate, precuneus, parietal, occipital and right temporal regions ↓ NDI left frontal, cingulate and paracingulate PD‐MCI vs. PD‐NC: ↓ ODI right frontal and caudate PD‐NC vs. HC: ↓ ODI cingulate, paracingulate, SMA, right precuneus No cortical thickness difference	↓ ODI in right frontal area associated with lower MOCA scores
Bai et al^29^	82 Early PD (Hoehn‐Yahr ≤2) 67 Moderate‐ late PD (Hoehn‐Yahr >2) 76 HC	Two‐shells (*b* = 1000, *b* = 2000) Cross‐sectional GBSS	Whole cortex	Widespread ↓ ODI in both early and late PD vs. HC. Late vs. early PD: ↓ ODI temporal and occipital lobes ↓ NDI mainly frontal/cingulate in both early and late PD vs. controls Cortical thinning in right fusiform in both PD groups vs. HC	↓ ODI (in areas differing between early vs. late PD) correlated with UPDRS‐III motor scores and global composite severity score
Mitchell et al^31^	37 PD 11 MSA‐P 17 PSP 21 HC	Three shells (*b* = 1000, *b* = 2000, *b* = 3000) Longitudinal region of interest analysis (1 yr follow‐up)	13 deep regions: SN, putamen, caudate, globus pallidus, subthalamic nucleus, red nucleus, thalamus, pedunculopontine nucleus, dentate nucleus, cerebellar lobules V–VI and vermis	Mild ↓ NDI in substantia nigra and putamen in PD vs. HC Less extensive changes than atypical syndromes No longitudinal changes in gray matter in PD	↑ FWF in substantia nigra predicted UPDRS‐III score across all parkinsonian patient groups
Bower et al.^32^	46 PD (28 tremor‐dominant, 18 PIGD)	One shell (*b* = 1000) Longitudinal region of interest analysis of FWF (2 yr follow up)	13 deep regions: subthalamic nucleus, red nucleus, thalamus, pedunculopontine nucleus, dentate nucleus, cerebellar lobules V–VI and vermis	No baseline between group differences ↑ FWF in PIGD compared to tremor‐dominant PD patients in putamen, globus pallidus and cerebellar lobule V over 2 yr	No correlation overall between FWF percentage change and UPDRS‐III change over 2 yr
DLB					
Mak et al.^33^	57 LBD (14 MCI‐LB, 43 DLB) 57 HC	Three shells (*b* = 500, *b* = 1000, *b* = 2000) Cross‐sectional region of interest analysis	43 cortical ROIs (Mayo Clinic Adult Lifespan Template atlas)	Widespread ↑ FWF/↓ tNDI and ↓ ODI in posterior regions in LBD vs. HC	↑ FWF (in regions showing differences between DLB vs. HC) weak correlation with UPDRS‐III No associations with NDI or ODI and motor or cognitive measures
Chiu et al.^34^	23 LBD (20 DLB, 3 MCI‐LB) 20 HC	Longitudinal region of interest analysis of FWF (up to 2 yr follow‐up)	43 cortical ROIs (Mayo Clinic Adult Lifespan Template atlas)	↑ FWF across multiple cortical and subcortical regions, more pronounced in temporal lobes in LBD only	FWF rate of change in right amygdala correlated with UPDRS‐III and in left inferior frontal operculum with MOCA

Abbreviations: PD, Parkinson's disease; HC, healthy controls; GBSS, gray matter based spatial statistics; FWF, free‐water fraction; NDI, neurite density index; ODI, orientation dispersion index; UPDRS, Unified Parkinson's Disease Rating Scale; yr, year; PD‐MCI, Parkinson's disease with cognitive impairment; PD‐NC, Parkinson's disease with normal cognition; SMA, spinal muscular atrophy; MOCA, Montreal Cognitive Assessment; MSA‐P, multiple system atrophy‐parkinsonian type; PSP, progressive supranuclear palsy; SN, substantia nigra; PIGD, postural instability and gait difficulties subtype of PD; DLB Dementia with Lewy bodies; LBD, Lewy body dementia; ROIs, regions of interest; MCI‐LB, mild cognitive impairment with Lewy bodies; tNDI, tissue‐adjusted neurite density index.

Critically, no study has yet comprehensively characterized gray matter microstructure across the continuum of Lewy body diseases or systematically examined how it relates to disease severity, Alzheimer's disease co‐pathology or longitudinal progression. Preliminary evidence suggests that progressive FWF increases may track disease progression, particularly for motor symptoms,^31^, ^34^ but this is less clear for cognition.

Here, we comprehensively examine microstructural properties (FWF, NDI, and ODI) compared to macrostructural atrophy of cortical and subcortical gray matter across the spectrum of Lewy body diseases and examine their relationship with disease severity, Alzheimer's disease co‐pathology, and clinical progression over time. First, we examine between‐group differences between cognitively impaired patients (LBD), PD with intact cognition (PD‐NC), and controls. Next, we evaluate associations with motor and cognitive severity and plasma p‐tau217. Finally, we assess whether NODDI‐derived measures show longitudinal change in a subset of baseline cognitively unimpaired PD patients followed‐up over 3 years.

## Methods

### Participants

All participants were recruited to University College London (UCL) and provided written consent. Ethical approval was granted by the Queen Square Ethics Committee (15.LO.0476).

Participants were required to be 50 years or older, capable of informed consent and, for participants:

- with PD have a clinical PD diagnosis within 10 years, per Movement Disorder's Society (MDS) clinical diagnostic criteria^35^;
- with DLB have a clinical diagnosis of probable DLB per McKeith criteria^36^;
- with PDD have a clinical diagnosis of PD dementia per MDS criteria,^37^ or, where comprehensive neuropsychology not possible, clinical PD diagnosis per MDS clinical diagnostic criteria plus impaired function in activities of daily living because of cognitive impairment (assessed using the Functional Activities Questionnaire^38^ and MDS Unified PD Rating Scale [UPRDS] question 1.1), and Montreal Cognitive Assessment (MoCA) ≤26^37^;
- with PD mild cognitive impairment (PD‐MCI) have a clinical PD diagnosis per MDS criteria and persistent performance below 1.5 standard deviations in one test of two cognitive domains or two tests in one cognitive domain^39^;
- with MCI with Lewy bodies (MCI‐LB) have a diagnosis of probable MCI‐LB per consensus criteria^40^; and
- control participants are 50 to 81 years, without cognitive impairment (Mini‐Mental State Examination [MMSE] ≥25).

Exclusion criteria included confounding neurological or psychiatric disorders or contraindications to MRI.

Longitudinal imaging and clinical data were available in a subset of PD (n = 100) and control (n = 27) participants, followed‐up with the same imaging and clinical protocol at 18‐ and 36 months.^41^ PD participants developing dementia or MCI during follow‐up were classified as having poor cognitive outcomes. For all participants in the longitudinal cohort, imaging and clinical data from their last available visit was used, similar to previous work.^42^ Participants with a diagnosis of DLB, PDD, PD‐MCI and MCI‐LB were grouped as LBD, with the remaining PD participants classified as PD‐NC.

### Clinical Assessments

The study protocol is previously described.^41^, ^42^, ^43^ Briefly, global disease burden was assessed using MDS‐UPDRS total score,^44^ motor severity with UPDRS part‐III, and Timed Up and Go (TUG).^45^ Cognition was assessed with MMSE^46^ and MoCA^47^ as global measures and with two tests per cognitive domain (Supplementary Methods). A composite cognitive score was calculated as the averaged z‐score of MoCA plus one task per cognitive domain.^43^ Levodopa equivalent daily doses (LEDD) were calculated for PD‐NC and LBD participants.^48^

### Plasma Collection and Processing

A total of 30 mL of blood was collected in polypropylene EDTA tubes. Samples were centrifuged and stored at −80°C. P‐tau217 concentration was measured using the AlzPath Simoa HD‐X p‐Tau217 Advantage‐PLUS kit (ALZPath/Quanterix, USA) by technicians blinded to clinical data. Measurements were performed in a single batch of reagents and were above the assay's limit detection. A total of 155 participants had samples available (66 LBD, 62 PD‐NC, and 27 controls) at a single time point and not included in longitudinal analyses.

### Image Acquisition and Preprocessing

All data were acquired on the same 3 T Siemens Prisma scanner with same acquisition parameters. DWI: b0 (Anterior‐Posterior (AP) and Posterior‐Anterior (PA)), *b* = 50s/mm^2^ 17 directions, *b* = 300 s/mm^2^ 8 directions, *b* = 1000s/mm^2^ 64 directions, b = 2000s/mm^2^ 64 directions, 2 × 2 × 2 mm isotropic voxels, pulse repetition time (TR) = 3260 ms, echo time (TE) = 58 ms, 72 slices, and acceleration factor = 2. Three dimensional magnetization prepared rapid gradient echo: TE = 3.34 ms, TR = 2530 ms, TI = 1100 ms, flip angle = 7°, 1 × 1 × 1 mm isotropic voxels, and acceleration factor = 2.

Raw image data were visually inspected by raters blinded to clinical data. Scans with artefacts in 15 or more volumes were excluded (n = 3 PD‐NC, n = 2 LBD excluded). MRI quality metrics were extracted from T1‐weighted images using MRIQC and compared between groups (Fig. S1). DWI images were preprocessed using standard pipelines in MRtrix3.0^49^ including denoising,^50^ removal of Gibbs artefacts,^51^ eddy‐current, susceptibility distortion and motion,^52^ and bias field correction.^53^ Raw T1‐weighted images were co‐registered to corresponding DWI using rigid and affine registration in NiftyReg^54^, and registrations visually assessed for alignment. A brain mask was calculated using Synthstrip^55^ and transformed to DWI‐space. For cortical thickness, FreeSurfer v6.0 pipeline was used with default parameters.

### NODDI Calculation

The NODDI model was fitted using accelerated microstructure imaging via convex optimization (AMICO) in Python^56^ with gray matter specific diffusion parameter 1.3 μm/ms within the previously calculated brain mask, to produce voxel‐wise FWF, ODI, and NDI maps. To avoid biased estimates because of CSF partial volume, we calculated tissue‐weighted maps (tODI and tNDI) by voxel‐wise multiplying each map with the tissue‐fraction map (1‐FWF).^57^ As is standard practice, we did not apply tissue‐weighting correction for FWF, which is a direct measure of isotropic free water.^57^ Voxel‐wise tODI, tNDI, FWF, and cortical thickness maps were projected to an FSLR32k surface using an algorithm weighted toward cortical mid‐thickness,^20^ to enable surface‐based comparisons between groups.

### Statistical Analysis

Demographics and clinical characteristics were compared between groups using analysis of variance (ANOVA) for normally distributed and Kruskal‐Wallis for non‐normally distributed variables (post hoc Tukey and Dunn, respectively), and Shapiro‐Wilks and visual inspection used to assess normality. Repeated‐measures ANOVA were used to assess group differences longitudinally.

Surface maps (FWF, tODI, tNDI, and cortical thickness) were averaged across 200 cortical (Schaeffer parcellation^58^) and 32 subcortical regions (Tian parcellation^59^) and a mean value per region calculated. For tODI and tNDI, these were weighted by the regional mean tissue fraction,^57^ as implemented in https://github.com/tdveale/TissueWeightedMean. Cross‐sectional group comparisons (LBD vs. healthy controls [HC], PD‐NC vs. HC, and LBD vs. PD‐NC) were performed using general linear models with age and sex as covariates and false discovery rate (FDR) correction for multiple comparisons, in BrainStat^60^ for cortical and statsmodels for subcortical regions.

Longitudinal comparisons were performed between PD good and PD poor cognitive outcomes using general linear models with baseline age, sex, and time‐between‐scans as covariates, time × group interaction as variable‐of‐interest, and a random intercept and slope per participant, FDR‐correcting for multiple comparisons. Correlations with disease severity metrics were performed using the same general linear models and covariates across all disease participants (excluding controls).

Sample size requirements for hypothetical disease modifying trials were calculated using simulation‐based power calculations of subject‐level longitudinal slopes, equivalent to testing treatment × time interaction under linear mixed‐effects models. Cognitive change was modelled linearly at baseline, 18‐, and 36‐ months. Variance components were empirically derived from the region with largest effect size (partial *r*). Simulated datasets mirrored this design. Treatment effects were defined as proportional reductions in excess cognitive decline observed between PD poor versus PD good outcomes (derived from observed combined cognitive scores in those patients), reflecting treatment‐induced differences in longitudinal slope. For each candidate sample size, 500 simulated trials were generated, mixed models refitted, and slopes compared between groups. The minimum per‐arm sample size achieving 80% power at α = 0.05 is reported.

### Data Sharing

Processing and analysis code and anonymized group‐level data will be available on publication through https://github.com/AngelikaZa/GM_Microstructure. Individual‐level and raw imaging data may be shared on reasonable request to the corresponding author.

## Results

We included 243 total participants: 197 across the Lewy body diseases spectrum, including 88 LBD (34 DLB, 34 PDD, 17 PD‐MCI, and 3 MCI‐LB); 109 PD‐NC; and 46 controls. Demographic and clinical information is seen in Table 2. Image quality did not significantly differ between groups (Fig. S1).

**TABLE 2:** Demographics and clinical characteristics in both the cross‐sectional and longitudinal cohorts

Characteristic	HC n = 46	PD‐NC n = 109	LBD n = 88	*P*‐value
Cross‐sectional cohort				
Age	67.2 (8.0)	64.0 (7.1)	70.9 (7.0)	**0.029** ^a^, ^b^, ^c^
Male (%)	23 (50.0)	58 (53.2)	72 (81.8)	**<0.001** ^a^, ^c^
Right‐handed (%)	40 (86.9)	102 (93.6)	77 (87.5)	0.353
Years of education	17.5 (3.0)	16.8 (2.5)	16.4 (3.4)	0.185
MOCA	27.9 (2.5)	28.4 (1.6)	23.4 (5.1)	**<0.001** ^a^, ^c^
MMSE	28.9 (1.5)	29.2 (1.1)	26.7 (3.4)	**<0.001** ^a^, ^c^
Composite cognitive score	–	−0.11 (0.65)	−1.89 (1.84)	**<0.001**
Disease duration	–	4.4 (2.7)	4.8 (4.5)	0.288
Duration of cognitive impairment	–	–	2.1 (1.5)	–
UPDRS total	–	45.4 (19.4)	66.9 (30.9)	**<0.001**
UPDRS part III	–	24.9 (19.4)	32.5 (17.4)	**0.003**
Timed Up and GO (seconds)	–	15.9 (7.3)	9.7 (4.2)	0.296
Functional Assessments Questionnaire	–	2.2 (2.2)	8.9 (7.1)	**<0.001**
CAF	–	2.4 (2.9)	5.1 (3.7)	**0.018**
One Day Fluctuations Questionnaire	–	1.4 (2.9)	5.1 (3.6)	**0.002**
LEDD		520.7 (368.3)	525.0 (258.7)	0.458
UMPDHQ		0.8 (1.6)	2.8 (3.3)	**<0.001**
RBDSQ		5.2 (3.0)	6.5 (4.1)	**<0.001**
Longitudinal cohort at baseline	HC (n = 27)	PD good outcomes (n = 69)	PD poor outcomes n = 31	
Age	65.9 (9.2)	62.4 (7.0)	68.4 (8.5)	**0.004** ^d^, ^e^
Male (%)	13 (48.1)	31 (44.9)	23 (74.2)	**0.022** ^d^
Right‐handed (%)	24 (88.9)	64 (92.7)	27 (87.1)	0.489
Years of education	18.0 (2.2)	16.9 (2.6)	17.5 (2.9)	0.203
MOCA	28.9 (1.3)	28.5 (1.4)	26.5 (3.3)	**<0.001** ^d^, ^e^
MMSE	29.2 (1.0)	29.1 (1.1)	28.4 (1.5)	**0.018** ^d^, ^e^
Composite cognitive score	0.05 (0.6)	−0.01 (0.6)	−0.93 (0.9)	**<0.001** ^d^, ^e^
Disease duration	–	4.1 (2.3)	4.9 (3.4)	0.209
UPDRS total	–	41.4 (18.7)	55.6 (32.9)	**0.007**
UPDRS part III	–	19.5 (9.5)	26.6 (18.8)	**0.042**
LEDD	–	451.8 (271.4)	484.3 (231.7)	0.195
UMPDHQ	–	0.7 (1.9)	1.1 (2.2)	0.164
RBDSQ	–	4.1 (2.5)	4.5 (2.5)	0.224

*Note*: All results shown as mean (standard deviation) except otherwise indicated. *P*‐values presented show overall differences between the three groups. Bold signifies differences between LBD and PD.

Abbreviations: HC, controls; PD‐NC, Parkinson's with normal cognition; LBD, Lewy body dementias; MOCA, Montreal Cognitive assessment; MMSE, Mini‐Mental State Examination; UPDRS, Movement Disorders Society Unified Parkinson's Disease Rating Scale; CAF, Clinician Assessment of Fluctuations; LEDD, levodopa equivalent daily dose; UMPDHQ, University of Miami PD Hallucinations Questionnaire; RBDSQ, Rapid Eye Movement Sleep Behavior Disorder Sleep Questionnaire; PD, Parkinson's disease.

^a^ Difference between LBD‐HC.

^b^ Difference between PD‐HC.

^c^ Difference between LBD‐PD.

^d^ Difference between PD poor vs. PD good outcomes.

^e^ Difference between PD poor outcomes and controls.

LBD subgroups did not significantly differ in demographic or clinical characteristics, other than lower composite cognitive scores in DLB and higher overall disease duration in PD‐MCI and PDD participants, as expected (Table S1).

A subset of 100 PD and 27 controls underwent longitudinal clinical and imaging assessments at 18‐ (session 1) and 36‐months (session 2). PD participants were baseline cognitively intact, but 31 developed MCI or dementia during follow‐up (PD poor outcomes). Our longitudinal cohort is previously described.^41^ Baseline group differences are presented in Table 2.

### Widespread Cortical and Subcortical Microstructural Alterations Are Seen, in the Absence of Atrophy, in LBD Compared to Controls and PD‐NC

**FIG. 1:**
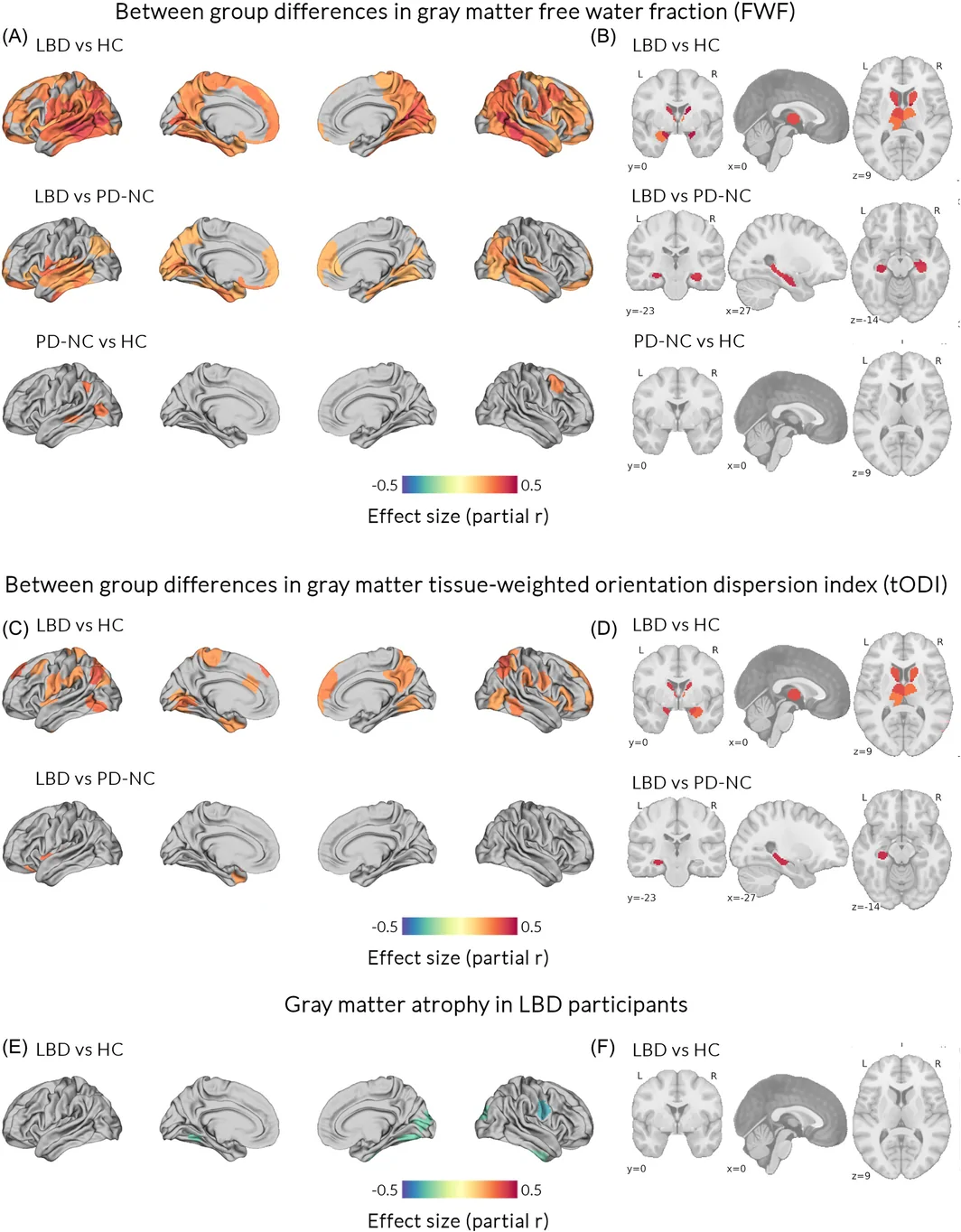
Microstructural gray matter changes measured using diffusion‐weighted imaging across the spectrum of Lewy body diseases. Between group differences were examined using general linear models with age and sex as nuisance covariates and FDR‐correction for multiple comparisons, in 197 participants across the spectrum of Lewy body diseases and 46 controls. We compared: LBD (n = 88) versus HC (n = 46), LBD versus PD‐NC (n = 109) and PD‐NC versus HC across 200 cortical and 32 subcortical regions. (**A**,**B**) FWF compared between groups in (**A**) cortical and (**B**) subcortical regions. (**C**,**D**) tODI compared between groups in cortical (**C**) and subcortical regions (**D**). (**E**,**F**) Cortical thickness (**E**) and subcortical gray matter volume (**F**) compared between groups. Color scale depicts effect size (blue colors indicate decreases in the examined group; red colors increases). Only statistically significant regions after multiple comparisons correction (FDR‐*P* < 0.05) are shown. There were no statistically significant group differences in tissue‐weighted neurite density index. LBD, Lewy body dementia patients; PD‐NC, Parkinson's disease patients with normal cognition; HC, age‐matched controls; FWF, free‐water fraction; tODI, tissue weighted‐orientation dispersion index; FDR, false discovery rate. [Color figure can be viewed at wileyonlinelibrary.com]

LBD participants showed widespread changes in gray matter microstructure. LBD showed widespread FWF increases in cortical (maximum effect size partial *r* = 0.453, FDR‐corrected *P*‐value *q* < 0.001) (Fig. 1A) and subcortical regions (*r* = 0.457, *q* < 0.001; right caudate) (Fig. 1B) compared to controls. LBD showed FWF increases compared to PD‐NC in pre‐frontal, occipital and medial temporal regions (*r* = 0.327, *q* < 0.001), and bilateral hippocampi (*r* = 0.595, *q* = 0.023; left posterior hippocampus).

LBD participants showed tODI increases compared to controls, with similar distribution as FWF but less widespread, with changes primarily within parietal, temporal and frontal lobes (*r* = 0.348, *q* = 0.008) (Fig. 1C), and basal ganglia (Fig. 1D). tODI increases were also seen in LBD compared to PD‐NC within right frontal and temporal regions (*r* = 0.313, *q* = 0.002) and left hippocampus (*r* = 0.586, *q* = 0.042).

There were no tNDI differences between groups. Additionally, there was minimal cortical (*r* = −0.270, *q* = 0.001) (Fig. 1E) and no subcortical atrophy in LBD compared to controls (Fig. 1F) and no atrophy differences between LBD and PD‐NC.

In contrast to changes seen in LBD participants, there were minimal differences in PD‐NC compared to controls, with FWF increases only in left temporo‐parietal and right frontal regions (*r* = 0.301, *q* = 0.032) and no tODI changes (Fig. 1A).

All between‐group differences remained unchanged when total intracranial volume or disease duration were included as nuisance covariates (Fig.  S2 and S3, respectively). There were no significant differences between LBD subgroups. Between group differences showed similar pattern when MCI participants were excluded (Fig. S4). Full subcortical results are seen in Tables S2–S4.

### Microstructural Alterations Are Associated with Cognitive but Not Motor Severity and with Plasma p‐tau217 Levels

**FIG. 2:**
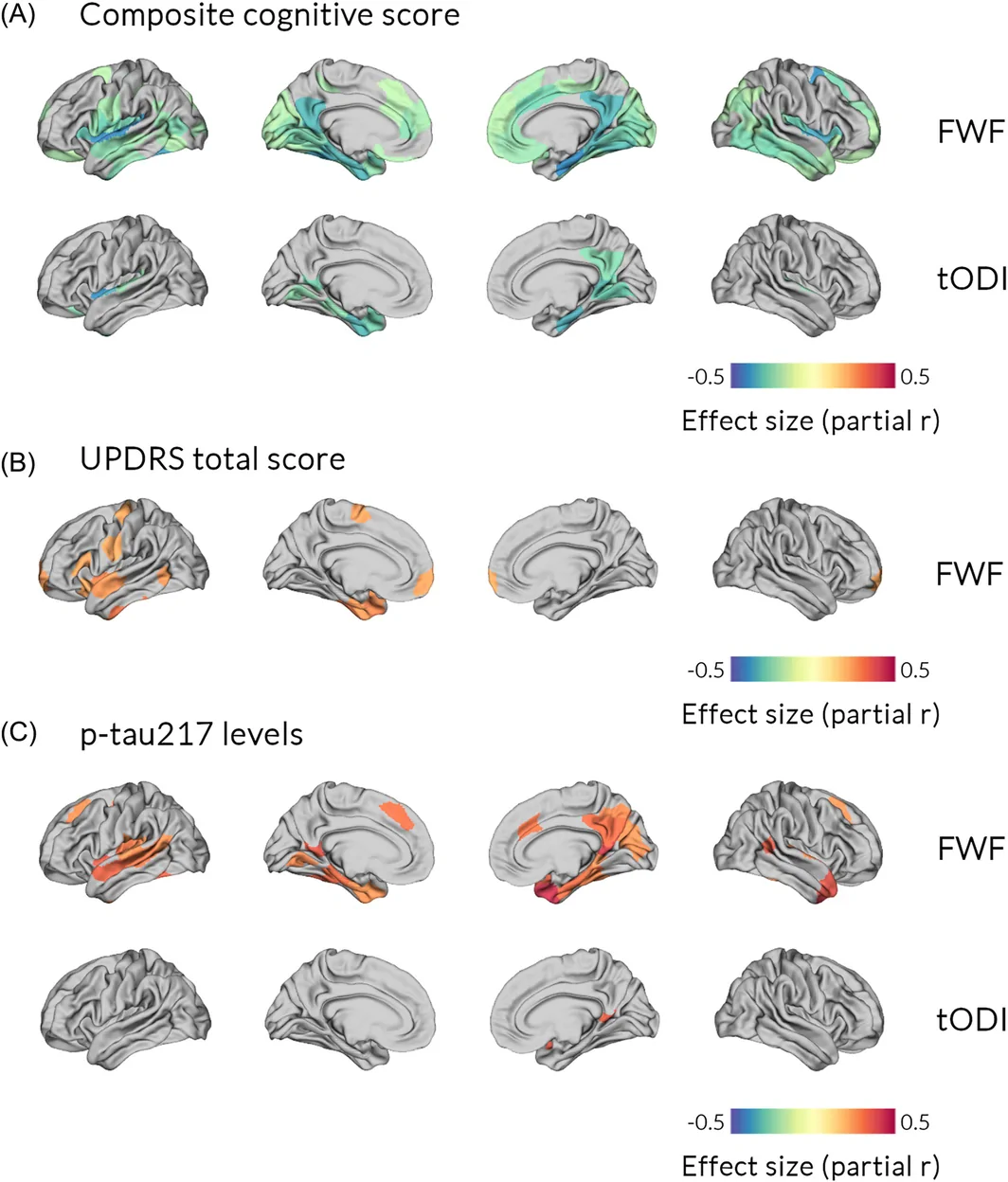
Microstructural gray matter alterations in Lewy body disease are correlated with cognitive, but not motor symptoms and with amyloid co‐pathology. (**A**) Increased cortical free‐water fraction (FWF) and tissue‐weighted orientation dispersion index (tODI) were correlated with lower composite cognitive scores in patients with Lewy body diseases (n = 197). (**B**) Increased FWF alone within prefrontal, temporal and parietal regions was associated with global disease severity in patients with Lewy body disease (n = 197), measured using the Movement Disorders Society Unified Parkinson's disease rating scale (UPDRS total score). (**C**) Increased FWF (top of subpanel) within temporal and parietal regions and increased tODI (bottom of subpanel) within the left temporal lobe were associated with increased plasma p‐tau217 levels, a plasma marker of brain β‐amyloid and tau co‐pathology burden. Analyses were performed across all participants with Lewy body diseases that had available p‐tau217 levels (n = 128). General linear models were used with age and sex as nuisance covariates and false discovery rate (FDR) correction for multiple comparisons across all participants with Lewy body diseases only (excluding controls). Color scale depicts effect size (blue colors indicates decreases in the examined group; red colors increases). Only statistically significant regions after multiple comparisons correction (FDR‐*P* < 0.05) are shown. [Color figure can be viewed at wileyonlinelibrary.com]

Increased cortical FWF, and to lesser extent tODI, was correlated with worse cognitive severity (composite cognitive score) (Fig. 2A) (MOCA and MMSE) (Fig. S5). Increased cortical FWF correlated with higher overall disease burden (total UPDRS) (Fig. 2B). Associations between FWF and cognitive severity metrics (but not total UPDRS) remained significant when adjusting for diagnostic group and disease duration (Figs. S6 and S7). For subcortical regions, only mean right hippocampus tODI signal was correlated with overall disease severity (total UPDRS).

FWF and tODI cortical, but not subcortical signal was correlated with plasma p‐tau217 levels, particularly within temporo‐parietal regions (FWF maximum *r* = 0.416, *q* < 0.001) (Fig. 2C), even adjusting for diagnostic group (Fig. S6) and disease duration (Fig. S7).

In contrast, cortical thickness and tNDI were not significantly correlated with any clinical or plasma measures.

### In PD Patients, Longitudinal Free‐Water Tracks Poor Cognitive Outcomes

Finally, we assessed whether gray matter microstructural integrity tracks disease progression longitudinally over 3‐years in 100 PD participants (n = 31 PD poor, *n* = 69 PD good outcomes).

**FIG. 3:**
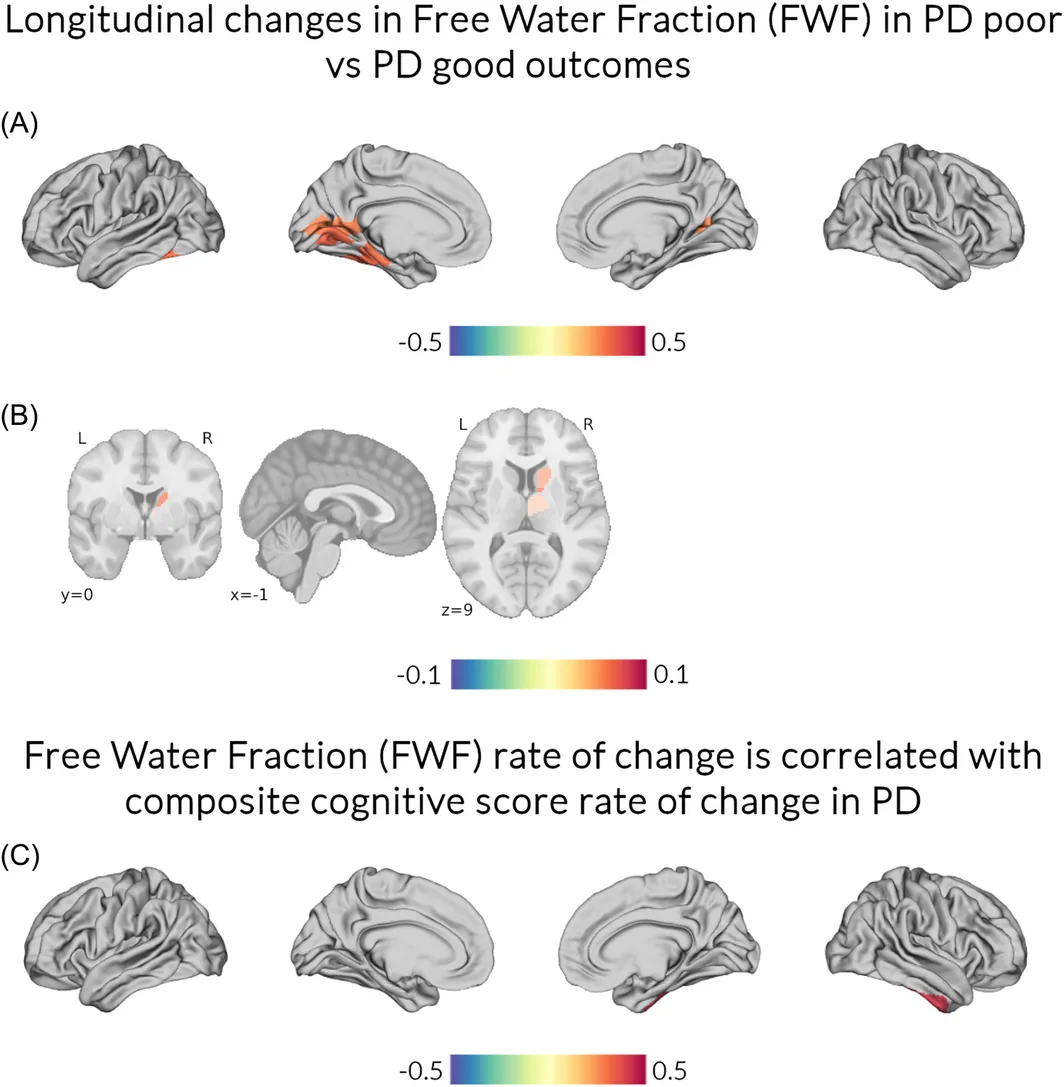
Free‐water fraction (FWF) tracks with longitudinal cognitive decline in Parkinson's disease (PD). General linear models were used to assess longitudinal differences in group × time interactions between patients with PD that developed mild cognitive impairment or dementia (PD poor outcomes, n = 31) compared to those with stable cognition (PD good outcomes, n = 69); age and sex as nuisance covariates, false discovery rate (FDR) correcting for multiple comparisons. Larger increases in FWF were seen longitudinally in PD poor compared to PD good outcomes in (**A**) cortical regions (temporal and occipital regions) and (**B**) subcortical regions (right basal ganglia and thalamus). (**C**) Rate of change in FWF was correlated with rate of change in composite cognitive scores within the right limbic temporal pole. Color scale depicts effect size (blue colors indicate decreases in the examined group; red colors increases). Only statistically significant regions after multiple comparisons correction (FDR‐*P* < 0.05) are shown. [Color figure can be viewed at wileyonlinelibrary.com]

PD poor outcomes showed higher longitudinal FWF increases compared to PD good outcomes within temporal regions (*r* = 0.257, *q* < 0.001) (Fig. 3A), right thalamus and caudate (Fig. 3B). There were no significant differences in tODI, tNDI, or cortical thickness. Similar results were seen between PD poor outcomes and controls (Fig. S8) with no differences between PD good outcomes and controls.

FWF rate‐of‐change within right limbic temporal pole correlated with longitudinal decline in composite cognitive scores (Fig. 3C) and MOCA (Fig. S9), but not motor or global severity. Plasma p‐tau levels were associated with increased FWF rate within the left frontal lobe, but no changes in other metrics.

Regions with maximum effect size per metric were used to calculate sample sizes for hypothetical clinical trials with combined cognitive scores as primary outcome measure (methodology in Table S5). Sample sizes were lowest for FWF (Table 3) with only 60 patients per arm needed to detect approximately10% slowing in cognitive decline.

**TABLE 3:** Estimated sample sizes per arm needed for a placebo‐controlled randomized clinical control trial of a hypothetical agent targeting cognition in Lewy body disease

Neuroimaging marker	Agent showing ~5% slowing in cognitive decline	Agent showing ~10% slowing in cognitive decline	Agent showing ~20% slowing in cognitive decline	Agent showing ~30% slowing in cognitive decline
Free water fraction	*n* = 220; power ~0.80	*n* = 60; power ~0.84	*n* = 20; power ~0.90	*n* = 10; power ~0.93
Tissue‐weighted ODI^a^	*n* = 330; power ~0.81	*n* = 80; power ~0.80	*n* = 25; power ~0.84	*n* = 15; power ~0.92
Cortical thickness^a^	*n* = 720; power ~0.81	*n* = 195; power ~0.80	*n* = 50; power ~0.83	*n* = 25; power ~0.83

*Note*: For all neuroimaging markers the first sample size per arm achieving power over 80% is reported and difference in combined cognitive scores is used as a primary cognitive outcome. Differences in slopes of cognitive decline were calculated based on combined cognitive score changes in PD patients with poor vs. good outcomes. The specific parameters used for the calculation of sample sizes per neuroimaging metric are presented in Table S4. Abbreviations: ODI, orientation dispersion index; PD, Parkinson's disease.

^a^ No statistically significant group differences were seen in these metrics, therefore, the region with the maximum effect size partial r was used for sample size calculations for these.

## Discussion

Here, we comprehensively examine gray matter microstructural integrity in 197 participants across the Lewy body disease spectrum, and 46 controls. We show widespread gray matter microstructural alterations in the absence of significant atrophy. LBD participants showed increased FWF and tODI compared to both controls and PD‐NC, with changes correlating with cognitive, but not motor severity and with Alzheimer's disease co‐pathology burden measured using plasma p‐tau217. Longitudinally, PD participants who later developed cognitive impairment showed faster FWF increases within temporal and subcortical regions. Together, these findings indicate that microstructural gray‐matter changes, particularly elevated FWF, reflect early, progressive gray matter pathology related to cognitive deterioration and Alzheimer's disease co‐pathology in Lewy body disease.

These measures reflect different microstructural properties. FWF refers to the fraction of diffusion signal attributable to extracellular water, not restricted by cell membranes or neurite structure (eg, CSF or enlarged interstitial space).^18^ Therefore, FWF increases may reflect extracellular fluid accumulation (eg, vasogenic edema), blood–brain barrier disruption, tissue atrophy below the threshold observable as gross structural changes, or neuroinflammation. FWF should be interpreted as an integrative, non‐specific marker of microstructural disruption rather than a direct measure of any single biological process. tODI reflects neurite angular variation within voxels, with increases suggesting more complex orientations, for example, dendrite branching.^18^, ^61^ Finally, tNDI estimates intra‐neurite volume fraction and serves as proxy for neurite density, with lower tNDI reflecting neurite loss.^18^, ^24^ The NODDI model relies on simplifying assumptions that may introduce bias when violated.^62^, ^63^ Particularly, using fixed diffusivity values for intra‐ and extra‐cellular compartments can lead to inaccurate metric estimation. Nevertheless, NODDI is one of the few diffusion models with substantial histological validation showing correspondence with microstructure.^64^, ^65^ However, these metrics should be interpreted cautiously as indices sensitive to rather than direct measures of biological processes.

Few studies have assessed gray matter microstructure in Lewy body diseases (Table 1). Increased FWF is shown separately in PD^17^ and DLB^33^, ^34^ compared to controls, largely relating to motor severity.^17^, ^31^, ^33^, ^34^ Consistent with this, we found widespread FWF increases in LBD patients compared to controls and PD‐NC. Our study extends previous work by including both cognitively intact and impaired patients with Lewy body diseases in one cohort. We show that frontal, temporal, and occipital changes in FWF correlate with cognitive, but not motor severity. Temporal FWF signal also increased with longitudinal cognitive impairment in PD, providing further evidence for microstructural gray matter alterations accompanying cognitive decline.

Additionally, we found increased tODI in LBD compared to controls and PD‐NC, correlating with cognitive severity, but not longitudinal progression. Mak et al^33^ recently described modest ODI reductions in DLB compared to controls, with reductions in right frontal lobe and caudate also described in a small study of PD‐MCI compared to PD‐NC.^29^ However, both studies had smaller sample sizes, did not use tissue‐correction for ODI and, crucially, had more severely affected cohorts (mean 3 points lower MMSE).^29^, ^33^ We used tissue‐weighting that reduces partial volume confounds, better isolating microstructural changes, whereas our incorporation of larger sample sizes across the disease spectrum and longitudinal data in a PD cohort could have enhanced sensitivity to subtle progression and cognition‐specific pathology. An alternative explanation is that differences in findings reflect differences in disease stage. NODDI‐derived tODI strongly correlates with dispersion in histologically validated studies,^64^, ^66^ mainly driven by sprawling dendritic processes and axonal dispersion in the gray matter. Axonal and dendritic structural dysfunction occur early in α‐synucleinopathies before overt axonal or neuronal loss: exogenous α‐synuclein in cell cultures induces dendritic spine impairment leading to dysmorphic dendrites and axons,^67^, ^68^, ^69^ overexpression leads to axonal swelling,^70^ and spine density increases are seen early within prefrontal cortex of transgenic mice.^71^ These processes could cause increased tODI at earlier dementia stages. Our study provides additional evidence that these changes may be detectable in vivo.

In contrast to widespread changes in FWF and tODI in LBD participants, we found no significant tNDI differences and minimal atrophy. Although tNDI and atrophy are both markers of neurodegeneration, atrophy can progress without measurable NDI change as NDI indexes microstructure of remaining tissue, rather than tissue already lost, and several studies have shown divergence between the two.^22^, ^28^, ^72^, ^73^, ^74^ Although neuronal loss eventually occurs in Lewy body diseases, preserved neuronal density at early stages is consistently seen despite evident functional alterations.^8^, ^68^, ^75^ Our findings, and previous neuroimaging studies showing heterogeneity in atrophy^10^, ^11^, ^12^, ^13^, ^14^, ^17^, ^76^ further support that metrics of overt neuronal loss are less sensitive in Lewy body diseases, particularly at earlier dementia stages, in contrast to Alzheimer's disease.^22^, ^25^, ^26^, ^73^

We did not find widespread group differences for atrophy or associations between cognitive severity and atrophy using conventional measures. This is consistent with previous work showing variability in cortical atrophy between LBD and PD‐NC^15^, ^76^ and for associations with cognition.^10^, ^12^, ^13^, ^14^, ^15^ For PD, cortical atrophy is even more inconsistent, with many studies showing little or no atrophy compared with controls and with cognitive severity.^15^, ^17^, ^30^, ^77^ Our finding of consistent group differences and associations between FWF and cognitive severity cross‐sectionally and over time, suggests that this measure is more sensitive to relevant tissue changes. These microstructural markers may serve as a stratification tool in therapeutic trials targeting cognitive decline in LBD, with smaller sample sizes required compared to cortical thickness.

We further observed higher FWF and tODI within temporo‐parietal regions correlated with plasma p‐tau217 levels, but this did not account for the full microstructural correlations seen in LBD patients. Although amyloid co‐pathology in Lewy body diseases is widespread, tau co‐pathology is generally localized within the temporal neocortex.^9^, ^78^ This corresponds with the regions showing correlations with p‐tau217 levels in our cohort. Increased tau burden in DLB is linked to worse cognition in vivo^42^, ^79^ and greater α‐synuclein cortical load at postmortem.^78^ Our findings, therefore, suggest that microstructural alterations observed in α‐synucleinopathies could reflect combined effects from both α‐synuclein and Alzheimer's disease co‐pathology.

Our study has limitations. Longitudinal data were available only for a subset of PD patients. NODDI metrics show good reproducibility, particularly within the gray matter,^80^, ^81^ and our cohort was enriched for cognitive impairment, with 31 participants developing dementia or MCI. Nevertheless, larger multicenter longitudinal studies could validate the reproducibility of FWF across different sites and scanners as a biomarker that tracks cognitive progression in Lewy body diseases and validate sample sizes for clinical trials. Although we applied tissue‐weighting to mitigate partial volume contamination it is not possible to fully disentangle extracellular free water from partial volume effects with CSF. FWF estimates may, therefore, be increased in patients because of subtle atrophy or motion. We show minimal atrophy in our cohort and no difference in MRI quality between groups, however, our results should be interpreted with caution and further validation is needed. We include patients across the Lewy body diseases spectrum, and LBD subgroups did not differ in clinical characteristics. However, the absence of subgroup differences should not be interpreted as biological equivalence between LBD subtypes, and we were not adequately powered to disentangle between‐subgroup differences, particularly within MCI subgroups. Although we examine the relationship between global Alzheimer's disease co‐pathology and microstructural gray matter alterations through plasma p‐tau217 levels, we did not have measures of abnormal α‐synuclein burden. Additionally, p‐tau217 levels were only available at a single time point, and we could not assess their longitudinal interaction with gray matter macro‐ and microstructure. Future research incorporating specific measures of pathological protein accumulation such as amyloid PET, tau PET, and α‐synuclein seed‐aggregation assays cross‐sectionally and longitudinally could disentangle causes of gray matter microstructural change in Lewy body diseases.

In summary, we comprehensively assess for the first time gray matter microstructure and macrostructure across the Lewy body disease spectrum cross‐sectionally and longitudinally. We show widespread increases in FWF and tissue‐weighted orientation dispersion in the absence of significant atrophy. This is consistent with extracellular expansion and dendritic disorganization rather than overt neuronal loss. These changes were specific to cognitive, but not motor severity and related to plasma p‐tau217. We also showed that FWF increased over time in PD patients who developed cognitive decline during follow‐up. Together, our results suggest that NODDI‐derived measures, particularly FWF, are sensitive to early and evolving gray matter changes and a potential neuroimaging biomarker of disease progression in LBD.

## Author Roles

(1) Research Project: A. Conception, B. Organization, C. Execution; (2) Statistical Analysis: A. Design, B. Execution, C. Review and Critique; (3) Manuscript Preparation: A. Writing of the First Draft, B. Review and Critique.

A.Z.:1A, 1B, 1C, 2A, 2B, 2C, 3A, 3B.

I.D.: 1C, 3B.

N.H.:1B, 1C, 3B.

R.B.: 1C, 3B.

G.T.: 1C, 3B.

S.C.: 1C, 3B.

A.J.H.: 1C, 3B.

E.V.: 1C, 3B.

H.Z.: 1C, 3B.

R.S.W.: 1A, 1B, 1C, 2C, 3B.

## Financial Disclosures and Conflicts of Interest

R.S.W. has received honoraria from GE Healthcare, Bial, Omnix Pharma, and Britannia; and consultancy fees from Therakind and Accenture and UCL Partners. H.Z. has served at scientific advisory boards and/or as a consultant for AbbVie, Acumen, Alector, Alzinova, ALZPath, Annexon, Apellis, Artery Therapeutics, AZTherapies, Cognito Therapeutics, CogRx, Denali, Eisai, Merry Life, Nervgen, Novo Nordisk, Optoceutics, Passage Bio, Pinteon Therapeutics, Prothena, Red Abbey Labs, reMYND, Roche, Samumed, Siemens Healthineers, Triplet Therapeutics, and Wave; has given lectures in symposia sponsored by Alzecure, Biogen, Cellectricon, Fujirebio, Lilly, and Roche; and is a co‐founder of Brain Biomarker Solutions in Gothenburg AB (BBS), which is a part of the GU Ventures Incubator Program (outside submitted work).

## Supporting information


**Table S1.** Clinical characteristics in subgroups of Lewy Body Dementia patients.
**Table S2.** Subcortical region of interest analyses for Free Water Fraction (FWF).
**Table S3.** Subcortical region of interest analyses for tissue‐weighted Orientation Dispersion Index (ODI).
**Table S4.** Subcortical region of interest analyses for tissue‐weighted Neurite Density Index (tNDI).
**Table S5.** Parameter estimation for Sample size calculations.
**Figure S1.** Image quality metrics in our cohort.
**Figure S2.** Between group differences in grey matter microstructure adjusting for total intracranial volume.
**Figure S3.** Between group differences in grey matter microstructure adjusting for disease duration.
**Figure S4.** Between group differences in grey matter microstructure including only patients with dementia for LBD comparisons excluding mild cognitive impairment (MCI).
**Figure S5.** Changes in microstructural measures in relation to MMSE and MOCA.
**Figure S6.** Changes in microstructural measures in relation to disease severity and p‐tau217 levels, adjusting for diagnostic group.
**Figure S7.** Changes in microstructural measures in relation to disease severity and p‐tau217 levels, adjusting for diagnostic group and disease duration.
**Figure S8.** Longitudinal changes in Free‐water fraction between PD‐poor outcomes and controls.
**Figure S9.** Longitudinal changes in Free‐water fraction in relation to MOCA.

## Data Availability

The data that support the findings of this study are available on request from the corresponding author. The data are not publicly available due to privacy or ethical restrictions.
