# Supplementary material for: Gray Matter Microstructure Measured Using Diffusion Imaging as a Biomarker of Severity in Lewy Body Diseases
Source: Mov Disord. 2026 May 25;41(9):2310–24. doi: 10.1002/mds.70355 (PMC13602283; doi:10.1002/mds.70355)
Supplement: Supplementary file 1 [file MDS-41-2310-s001.docx]

# Supplementary Material: Grey matter microstructure measured using diffusion imaging as a biomarker of severity in Lewy body diseases

Dr Angeliki Zarkali^1,2,3,4^, Ms Ivelina Dobreva^1^, Ms Naomi Hannaway^1^, Dr Rohan Bhome^1^, Dr George Thomas^1^, Ms Steffani Carreira^1^, Dr Amanda J Heslegrave^5^, Ms Elena Veleva^5^, Prof Henrik Zetterberg^1,5^, Prof Rimona S Weil^1,2,3,4^

## Contents

[Supplementary Material: Grey matter microstructure measured using diffusion imaging as a biomarker of severity in Lewy body diseases	1](#_Toc225092568)

[Contents	1](#_Toc225092569)

[Supplementary Methods	2](#_Toc225092570)

[Supplementary Figure 1. Image quality metrics in our cohort	2](#_Toc225092571)

[Supplementary Table 1. Clinical characteristics in subgroups of Lewy Body Dementia patients	5](#_Toc225092572)

[Supplementary Table 2. Subcortical region of interest analyses for Free Water Fraction (FWF).	6](#_Toc225092573)

[Supplementary Table 3. Subcortical region of interest analyses for tissue-weighted Orientation Dispersion Index (ODI).	7](#_Toc225092574)

[Supplementary Table 4. Subcortical region of interest analyses for tissue-weighted Neurite Density Index (tNDI).	8](#_Toc225092575)

[Supplementary Figure 2. Between group differences in grey matter microstructure adjusting for total intracranial volume	9](#_Toc225092576)

[Supplementary Figure 3. Between group differences in grey matter microstructure adjusting for disease duration	10](#_Toc225092577)

[Supplementary Figure 4. Between group differences in grey matter microstructure including only demented patients (excluding mild cognitive impairment (MCI).	12](#_Toc225092578)

[Supplementary Figure 5. Changes in microstructural measures in relation to MMSE and MOCA	13](#_Toc225092579)

[Supplementary Figure 6. Changes in microstructural measures in relation to disease severity and p-tau217 levels, adjusting for diagnostic group	14](#_Toc225092580)

[Supplementary Figure 7. Changes in microstructural measures in relation to disease severity and p-tau217 levels, adjusting for diagnostic group and disease duration	15](#_Toc225092581)

[Supplementary Figure 8. Longitudinal changes in Free-water fraction between PD-poor outcomes and controls	16](#_Toc225092582)

[Supplementary Figure 9. Longitudinal changes in Free-water fraction in relation to MOCA	16](#_Toc225092583)

[Supplementary Table 5. Parameter estimation for Sample size calculations	16](#_Toc225092584)

[URLs:	17](#_Toc225092585)

[References	18](#_Toc225092586)

## Supplementary Methods

**Details of clinical assessments used**

All participants completed two tasks per cognitive domain as well as MMSE^1^ and MoCA^2^. These included:

- *Attention* (Stroop colour naming^3^ and Digit Span Backwards^4^),
- *Executive function* (Category Fluency^5^ and Stroop interference^3^)
- *Language* (Letter fluency^5^, and Graded Naming task^6^)
- *Memory* (Word recognition^6^ and Logical Memory delayed^4^) and
- *Visuospatial function* (Hooper Test of Visual Organisation ^7^ and Benton Judgement of Line Orientation^8^).

A composite cognitive score was calculated per participant as the averaged z-score of the MoCA plus one task per cognitive domain, as in our previous work^9^.

Cognitive fluctuations were assessed using the Clinician assessment of fluctuations (CAF) and One Day Fluctuations scale^10^. Impairment in daily functioning was assessed using the Functional Activities Questionnaire^11^, sleep using the Rapid Eye Movement Behaviour Disorder Sleep Questionnaire (RBDSQ)^12^ and visual hallucinations using the University of Miami PD Hallucinations Questionnaire (UMPDHQ)^13^. Levodopa equivalent daily doses (LEDD) were calculated for all PD-NC and LBD participants^14^.

For the classification of patients as Parkinson’s Dementia (PDD) we required a clinical diagnosis of PD dementia per MDS criteria^15^, or, where comprehensive neuropsychology was not possible, a clinical PD diagnosis per MDS clinical diagnostic criteria^16^ plus impaired function in activities of daily living due to cognitive impairment (assessed using the Functional Activities Questionnaire^11^ and MDS Unified PD Rating Scale (UPRDS) Question 1.1)^17^ and Montreal Cognitive Assessment (MoCA) ≤26. This threshold was selected to maximise sensitivity to early cognitive changes, given that the main aim of our study was to identify correlates of early cognitive decline. Importantly, cognitive impairment in this study was not defined by MoCA alone but required concurrent functional decline (FAQ, MDS-UPDRS 1.1) and clinical diagnostic criteria. As such, the inclusion of participants with MoCA scores between 20-26 is unlikely to reflect false positives but rather milder yet clinically meaningful cognitive impairment.

Control participants were excluded if they had an MMSE score of ≤25. This was chosen as a screening tool for cognition to ensure comparability with previous work^18–20^ and reduce participant burden.

## Supplementary Figure 1. Image quality metrics in our cohort

Image quality metrics per participant were extracted from T1-weighted imaging using MRIQC version 23.1.0. T1-weighted imaging was used to extract quality metrics as this was used to define the cortical ribbon and the cortical and subcortical parcellations.

Signal to noise ratio (SNR) both **total (A)** and within the **grey matter (B)** did not significantly differ between groups (Total SNR: p=0.873 ; GM SNR: p=0.601; higher values indicating higher data quality). Total and grey matter SNR were also not significantly correlated with cognition as measured by the combined cognitive score (Total SNR: Spearman r=0.105, p=0.166; grey matter SNR: r=0.093, p=0.224), with age (Total SNR: r=-0.131, p=0.05, grey matter SNR: r=-0.065, p=0.328),

Foreground-Background Energy Ratio (FBER) **(C)**, an information-based theory metric with lower values indicating higher data quality, also did not significantly differ between groups (p=0.099) and neither did it correlated with cognition (r=-0.043, p=0.576) or age (r=-0.040, p=0.542),

**A**

**B**

**C**

## Supplementary Table 1. Clinical characteristics in subgroups of Lewy Body Dementia patients

| **Characteristic** | **Parkinson’s with cognitive impairment (PD-MCI)**  **n=17** | **Mild cognitive impairment with Lewy Bodies (LB-MCI)**  **n=3** | **Parkinson’s dementia (PDD)**  **n=34** | **Dementia with Lewy Bodies**  **(DLB)**  **n=34** | **p-value** |
| --- | --- | --- | --- | --- | --- |
| **Age** | 67.3 (8.0) | 70.4 (2.9) | 71.4 (7.0) | 72.2 (6.3) | 0.136 |
| **Male (%)** | 15 (88.2) | 3 (100) | 23 (67.7) | 31 (91.2) | 0.052 |
| **Right-handed (%)** | 14 (82.4) | 2 (66.7) | 30 (88.2) | 31 (91.2) | 0.436 |
| **Years of education** | 16.4 (2.4) | 13.8 (1.8) | 16.8 (3.8) | 16.3 (3.5) | 0.341 |
| **MOCA** | 25.6 (2.5) | 25.7 (2.5) | 23.6 (5.6) | 22.1 (5.5) | 0.127 |
| **MMSE** | 27.9 (2.0) | 27.7 (1.2) | 23.6 (3.5) | 25.5 (3.7) | 0.037* |
| **Composite cognitive score** | -1.2 (1.2) | -1.2 (0.5) | -1.0 (1.6) | -2.6 (1.9) | **0.015^a^** |
| **Disease duration** | 7.8 (5.1) | 0.3 (0.3) | 6.4 (4.7) | 2.2 (1.8) | **<0.001^a,b^** |
| **Duration of cognitive impairment** | 1.9 (1.3) | 0.4 (0.3) | 2.3 (1.1) | 2.2 (1.8) | 0.075 |
| **UPDRS total** | 57.2 (26.1) | 38.3 (27.0) | 73.7 (34.7) | 65.9 (28.9) | 0.088 |
| **UPDRS part 3** | 26.2 (14.1) | 16.0 (15.4) | 35.9 (18.1) | 32.7 (18.1) | 0.104 |
| **Timed Up and GO test (seconds)** | 9.4 (4.9) | 8.7 (1.1) | 8.9 (3.9) | 9.7 (4.7) | 0.999 |
| **Functional Assessments Questionnaire** | 8.0 (7.3) | 6.0 (4.6) | 8.0 (7.8) | 9.6 (6.8) | 0.821 |
| **LEDD** | 612.4 (355.5) | 410.0 (56.7) | 565.6 (246.3) | 263.2 (265.7) | **0.001^a^** |
| **UMPDHQ** | 1.9 (3.1) | 2.5 (3.5) | 2.8 (3.6) | 3.2 (1.6) | 0.510 |
| **RBDSQ** | 5.9 (3.8) | 8.5 (3.5) | 5.5 (3.8) | 7.6 (3.1) | 0.167 |
| ^*^ No posthoc between group differences.  ^a^: difference between PDD-LBD, ^b^: difference between PD-MCI and LB-MCI. In bold significant differences between subgroups. All results shown as mean (standard deviation) except otherwise indicated.  LEDD: Levodopa Equivalent Daily Dose; MMSE: Mini-Mental State Examination; MOCA: Montreal Cognitive assessment; RBDSQ: REM Sleep Behaviour Disorder Sleep Questionnaire; UMPDHQ: University of Miami PD Hallucinations Questionnaire; UPDRS: Movement Disorders Society Unified Parkinson’s Disease Rating Scale. | | | | | |

## Supplementary Table 2. Subcortical region of interest analyses for Free Water Fraction (FWF).

| **Region label** | **LBD vs HC** | | | **PD-NC vs HC** | | | **LBD vs PD-NC** | | |
| --- | --- | --- | --- | --- | --- | --- | --- | --- | --- |
|  | **β** | **p** | **q** | **β** | **p** | **q** | **β** | **p** | **q** |
| aHIP-rh | **0.027** | **0.002** | **0.016** | 0.016 | 0.198 | 1.000 | **0.046** | **0.001** | **0.022** |
| pHIP-rh | **0.044** | **0.000** | **0.001** | 0.021 | 0.241 | 1.000 | **0.056** | **0.001** | **0.022** |
| lAMY-rh | 0.016 | 0.015 | 0.082 | 0.022 | 0.022 | 0.424 | 0.009 | 0.426 | 1.000 |
| mAMY-rh | **0.038** | **0.000** | **0.000** | 0.037 | 0.012 | 0.290 | 0.036 | 0.010 | 0.143 |
| THA-DP-rh | 0.025 | 0.037 | 0.179 | 0.009 | 0.662 | 1.000 | 0.043 | 0.020 | 0.216 |
| THA-VP-rh | 0.005 | 0.502 | 1.000 | -0.007 | 0.536 | 1.000 | 0.008 | 0.384 | 1.000 |
| THA-VA-rh | **0.029** | **0.006** | **0.038** | 0.002 | 0.926 | 1.000 | 0.049 | 0.001 | 0.022 |
| THA-DA-rh | 0.040 | 0.012 | 0.070 | 0.011 | 0.696 | 1.000 | 0.055 | 0.031 | 0.274 |
| NAc-shell-rh | 0.001 | 0.915 | 1.000 | -0.009 | 0.513 | 1.000 | 0.011 | 0.281 | 1.000 |
| NAc-core-rh | 0.019 | 0.142 | 0.648 | 0.013 | 0.497 | 1.000 | 0.028 | 0.154 | 0.820 |
| pGP-rh | 0.002 | 0.536 | 1.000 | -0.001 | 0.921 | 1.000 | 0.002 | 0.711 | 1.000 |
| aGP-rh | 0.004 | 0.361 | 1.000 | 0.000 | 0.954 | 1.000 | 0.006 | 0.463 | 1.000 |
| aPUT-rh | 0.001 | 0.827 | 1.000 | 0.001 | 0.838 | 1.000 | -0.002 | 0.716 | 1.000 |
| pPUT-rh | 0.000 | 0.898 | 1.000 | 0.001 | 0.882 | 1.000 | -0.001 | 0.847 | 1.000 |
| aCAU-rh | **0.069** | **0.000** | **0.005** | 0.047 | 0.119 | 1.000 | 0.076 | 0.010 | 0.143 |
| pCAU-rh | **0.108** | **0.000** | **0.000** | 0.080 | 0.035 | 0.478 | 0.103 | 0.007 | 0.143 |
| aHIP-lh | **0.032** | **0.000** | **0.002** | 0.033 | 0.007 | 0.278 | 0.030 | 0.024 | 0.232 |
| pHIP-lh | **0.032** | **0.006** | **0.038** | -0.002 | 0.921 | 1.000 | **0.061** | **0.000** | **0.022** |
| lAMY-lh | **0.022** | **0.005** | **0.038** | 0.013 | 0.220 | 1.000 | 0.027 | 0.020 | 0.216 |
| mAMY-lh | **0.048** | **0.000** | **0.001** | 0.047 | 0.008 | 0.278 | 0.030 | 0.095 | 0.584 |
| THA-DP-lh | 0.032 | 0.015 | 0.082 | 0.024 | 0.273 | 1.000 | 0.019 | 0.338 | 1.000 |
| THA-VP-lh | 0.018 | 0.012 | 0.070 | 0.012 | 0.299 | 1.000 | 0.007 | 0.465 | 1.000 |
| THA-VA-lh | **0.046** | **0.000** | **0.003** | 0.041 | 0.050 | 0.596 | 0.039 | 0.049 | 0.389 |
| THA-DA-lh | 0.046 | 0.004 | 0.034 | 0.031 | 0.253 | 1.000 | 0.041 | 0.097 | 0.584 |
| NAc-shell-lh | 0.006 | 0.441 | 1.000 | -0.001 | 0.938 | 1.000 | 0.013 | 0.289 | 1.000 |
| NAc-core-lh | 0.040 | 0.016 | 0.082 | 0.028 | 0.262 | 1.000 | 0.047 | 0.074 | 0.543 |
| pGP-lh | 0.002 | 0.603 | 1.000 | 0.001 | 0.881 | 1.000 | 0.004 | 0.547 | 1.000 |
| aGP-lh | 0.005 | 0.239 | 0.996 | 0.002 | 0.827 | 1.000 | 0.007 | 0.353 | 1.000 |
| aPUT-lh | 0.004 | 0.204 | 0.891 | 0.008 | 0.190 | 1.000 | 0.003 | 0.590 | 1.000 |
| pPUT-lh | 0.002 | 0.456 | 1.000 | 0.001 | 0.817 | 1.000 | 0.002 | 0.659 | 1.000 |
| aCAU-lh | **0.093** | **0.000** | **0.002** | 0.089 | 0.033 | 0.478 | 0.065 | 0.087 | 0.584 |
| pCAU-rh | **0.113** | **0.000** | **0.001** | 0.128 | 0.009 | 0.278 | 0.064 | 0.140 | 0.788 |
| aHIP-lh | **0.027** | **0.002** | **0.016** | 0.016 | 0.198 | 1.000 | **0.046** | **0.001** | **0.022** |
| pHIP-lh | **0.044** | **0.000** | **0.001** | 0.021 | 0.241 | 1.000 | **0.056** | **0.001** | **0.022** |
| lAMY-lh | 0.016 | 0.015 | 0.082 | 0.022 | 0.022 | 0.424 | 0.009 | 0.426 | 1.000 |
| mAMY-lh | **0.038** | **0.000** | **0.000** | 0.037 | 0.012 | 0.290 | 0.036 | 0.010 | 0.143 |
| THA-DP-lh | 0.025 | 0.037 | 0.179 | 0.009 | 0.662 | 1.000 | 0.043 | 0.020 | 0.216 |
| THA-VP-lh | 0.005 | 0.502 | 1.000 | -0.007 | 0.536 | 1.000 | 0.008 | 0.384 | 1.000 |
| THA-VA-lh | **0.029** | **0.006** | **0.038** | 0.002 | 0.926 | 1.000 | **0.049** | **0.001** | **0.022** |
| THA-DA-lh | 0.040 | 0.012 | 0.070 | 0.011 | 0.696 | 1.000 | 0.055 | 0.031 | 0.274 |
| NAc-shell-lh | 0.001 | 0.915 | 1.000 | -0.009 | 0.513 | 1.000 | 0.011 | 0.281 | 1.000 |
| NAc-core-lh | 0.019 | 0.142 | 0.648 | 0.013 | 0.497 | 1.000 | 0.028 | 0.154 | 0.820 |
| pGP-lh | 0.002 | 0.536 | 1.000 | -0.001 | 0.921 | 1.000 | 0.002 | 0.711 | 1.000 |
| aGP-lh | 0.004 | 0.361 | 1.000 | 0.000 | 0.954 | 1.000 | 0.006 | 0.463 | 1.000 |
| aPUT-lh | 0.001 | 0.827 | 1.000 | 0.001 | 0.838 | 1.000 | -0.002 | 0.716 | 1.000 |
| pPUT-lh | 0.000 | 0.898 | 1.000 | 0.001 | 0.882 | 1.000 | -0.001 | 0.847 | 1.000 |
| aCAU-lh | **0.069** | **0.000** | **0.005** | 0.047 | 0.119 | 1.000 | 0.076 | 0.010 | 0.143 |
| pCAU-lh | **0.108** | **0.000** | **0.000** | 0.080 | 0.035 | 0.478 | 0.103 | 0.007 | 0.143 |

## Supplementary Table 3. Subcortical region of interest analyses for tissue-weighted Orientation Dispersion Index (ODI).

| **Region label** | **LBD vs HC** | | | **PD-NC vs HC** | | | **LBD vs PD-NC** | | |
| --- | --- | --- | --- | --- | --- | --- | --- | --- | --- |
|  | **β** | **p** | **q** | **β** | **p** | **q** | **β** | **p** | **q** |
| aHIP-rh | 0.018 | 0.037 | 0.110 | 0.007 | 0.349 | 1.000 | 0.016 | 0.035 | 0.479 |
| pHIP-rh | **0.001** | **0.004** | **0.013** | 0.021 | 0.065 | 0.896 | 0.018 | 0.162 | 0.915 |
| lAMY-rh | **0.002** | **0.005** | **0.015** | 0.010 | 0.283 | 1.000 | 0.007 | 0.403 | 1.000 |
| mAMY-rh | **0.000** | **0.002** | **0.005** | 0.014 | 0.172 | 1.000 | 0.015 | 0.119 | 0.759 |
| THA-DP-rh | 0.154 | 0.197 | 0.590 | 0.003 | 0.797 | 1.000 | 0.021 | 0.083 | 0.759 |
| THA-VP-rh | 0.119 | 0.166 | 0.498 | -0.001 | 0.849 | 1.000 | 0.015 | 0.034 | 0.479 |
| THA-VA-rh | **0.003** | **0.009** | **0.028** | 0.008 | 0.546 | 1.000 | 0.034 | 0.008 | 0.378 |
| THA-DA-rh | 0.013 | 0.028 | 0.085 | 0.017 | 0.448 | 1.000 | 0.036 | 0.085 | 0.759 |
| NAc-shell-rh | 0.315 | 0.338 | 1.000 | 0.003 | 0.771 | 1.000 | 0.005 | 0.565 | 1.000 |
| NAc-core-rh | 0.175 | 0.216 | 0.647 | 0.008 | 0.647 | 1.000 | 0.018 | 0.283 | 1.000 |
| pGP-rh | 0.035 | 0.056 | 0.168 | 0.001 | 0.903 | 1.000 | -0.015 | 0.114 | 0.759 |
| aGP-rh | 0.255 | 0.291 | 0.874 | 0.006 | 0.522 | 1.000 | -0.010 | 0.226 | 1.000 |
| aPUT-rh | 0.413 | 0.426 | 1.000 | -0.005 | 0.547 | 1.000 | -0.006 | 0.407 | 1.000 |
| pPUT-rh | 0.317 | 0.338 | 1.000 | -0.005 | 0.465 | 1.000 | -0.004 | 0.476 | 1.000 |
| aCAU-rh | **0.001** | **0.004** | **0.013** | 0.047 | 0.110 | 1.000 | 0.059 | 0.041 | 0.497 |
| pCAU-rh | **0.000** | **0.001** | **0.003** | 0.060 | 0.056 | 0.896 | 0.073 | 0.028 | 0.479 |
| aHIP-lh | 0.025 | 0.044 | 0.133 | 0.014 | 0.049 | 0.896 | 0.000 | 0.963 | 1.000 |
| pHIP-lh | **0.001** | **0.004** | **0.013** | 0.001 | 0.930 | 1.000 | **0.038** | **0.000** | **0.042** |
| lAMY-lh | 0.112 | 0.163 | 0.490 | 0.008 | 0.407 | 1.000 | -0.003 | 0.713 | 1.000 |
| mAMY-lh | **0.000** | **0.001** | **0.003** | 0.029 | 0.017 | 0.818 | 0.010 | 0.397 | 1.000 |
| THA-DP-lh | 0.134 | 0.179 | 0.537 | 0.020 | 0.212 | 1.000 | -0.008 | 0.571 | 1.000 |
| THA-VP-lh | **0.004** | **0.009** | **0.028** | 0.018 | 0.039 | 0.896 | 0.004 | 0.665 | 1.000 |
| THA-VA-lh | **0.000** | **0.002** | **0.006** | 0.028 | 0.129 | 1.000 | 0.042 | 0.018 | 0.479 |
| THA-DA-lh | **0.006** | **0.014** | **0.043** | 0.018 | 0.412 | 1.000 | 0.043 | 0.032 | 0.479 |
| NAc-shell-lh | **0.006** | **0.014** | **0.043** | 0.011 | 0.275 | 1.000 | 0.014 | 0.104 | 0.759 |
| NAc-core-lh | 0.029 | 0.049 | 0.147 | 0.017 | 0.398 | 1.000 | 0.034 | 0.107 | 0.759 |
| pGP-lh | 0.021 | 0.039 | 0.117 | -0.006 | 0.574 | 1.000 | -0.011 | 0.253 | 1.000 |
| aGP-lh | 0.245 | 0.291 | 0.872 | -0.003 | 0.738 | 1.000 | -0.003 | 0.639 | 1.000 |
| aPUT-lh | 0.750 | 0.750 | 1.000 | 0.000 | 0.951 | 1.000 | -0.008 | 0.269 | 1.000 |
| pPUT-lh | 0.081 | 0.124 | 0.371 | -0.007 | 0.352 | 1.000 | -0.010 | 0.119 | 0.759 |
| aCAU-lh | **0.000** | **0.002** | **0.007** | 0.082 | 0.034 | 0.896 | 0.040 | 0.236 | 1.000 |
| pCAU-rh | **0.000** | **0.001** | **0.003** | 0.111 | 0.012 | 0.818 | 0.058 | 0.149 | 0.896 |
| aHIP-lh | 0.018 | 0.037 | 0.110 | 0.007 | 0.349 | 1.000 | 0.016 | 0.035 | 0.479 |
| pHIP-lh | **0.001** | **0.004** | **0.013** | 0.021 | 0.065 | 0.896 | 0.018 | 0.162 | 0.915 |
| lAMY-lh | **0.002** | **0.005** | **0.015** | 0.010 | 0.283 | 1.000 | 0.007 | 0.403 | 1.000 |
| mAMY-lh | **0.000** | **0.002** | **0.005** | 0.014 | 0.172 | 1.000 | 0.015 | 0.119 | 0.759 |
| THA-DP-lh | 0.154 | 0.197 | 0.590 | 0.003 | 0.797 | 1.000 | 0.021 | 0.083 | 0.759 |
| THA-VP-lh | 0.119 | 0.166 | 0.498 | -0.001 | 0.849 | 1.000 | 0.015 | 0.034 | 0.479 |
| THA-VA-lh | **0.003** | **0.009** | **0.028** | 0.008 | 0.546 | 1.000 | 0.034 | 0.008 | 0.378 |
| THA-DA-lh | 0.013 | 0.028 | 0.085 | 0.017 | 0.448 | 1.000 | 0.036 | 0.085 | 0.759 |
| NAc-shell-lh | 0.315 | 0.338 | 1.000 | 0.003 | 0.771 | 1.000 | 0.005 | 0.565 | 1.000 |
| NAc-core-lh | 0.175 | 0.216 | 0.647 | 0.008 | 0.647 | 1.000 | 0.018 | 0.283 | 1.000 |
| pGP-lh | 0.035 | 0.056 | 0.168 | 0.001 | 0.903 | 1.000 | -0.015 | 0.114 | 0.759 |
| aGP-lh | 0.255 | 0.291 | 0.874 | 0.006 | 0.522 | 1.000 | -0.010 | 0.226 | 1.000 |
| aPUT-lh | 0.413 | 0.426 | 1.000 | -0.005 | 0.547 | 1.000 | -0.006 | 0.407 | 1.000 |
| pPUT-lh | 0.317 | 0.338 | 1.000 | -0.005 | 0.465 | 1.000 | -0.004 | 0.476 | 1.000 |
| aCAU-lh | **0.001** | **0.004** | **0.013** | 0.047 | 0.110 | 1.000 | 0.059 | 0.041 | 0.497 |
| pCAU-lh | **0.000** | **0.001** | **0.003** | 0.060 | 0.056 | 0.896 | 0.073 | 0.028 | 0.479 |

## Supplementary Table 4. Subcortical region of interest analyses for tissue-weighted Neurite Density Index (tNDI).

| **Region label** | **LBD vs HC** | | | **PD-NC vs HC** | | | **LBD vs PD-NC** | | |
| --- | --- | --- | --- | --- | --- | --- | --- | --- | --- |
|  | **β** | **p** | **q** | **β** | **p** | **q** | **β** | **p** | **q** |
| aHIP-rh | 0.012 | 0.032 | 0.513 | 0.016 | 0.141 | 0.348 | 0.013 | 0.142 | 0.569 |
| pHIP-rh | 0.011 | 0.044 | 0.569 | 0.006 | 0.418 | 0.581 | 0.014 | 0.077 | 0.426 |
| lAMY-rh | 0.006 | 0.076 | 0.659 | 0.026 | 0.004 | 0.059 | -0.015 | 0.070 | 0.426 |
| mAMY-rh | 0.021 | 0.006 | 0.307 | 0.034 | 0.020 | 0.202 | 0.006 | 0.667 | 0.847 |
| THA-DP-rh | 0.008 | 0.216 | 1.000 | 0.021 | 0.061 | 0.202 | -0.005 | 0.583 | 0.801 |
| THA-VP-rh | -0.002 | 0.620 | 1.000 | 0.008 | 0.292 | 0.513 | -0.016 | 0.014 | 0.222 |
| THA-VA-rh | -0.004 | 0.470 | 1.000 | 0.002 | 0.836 | 0.865 | -0.010 | 0.236 | 0.754 |
| THA-DA-rh | 0.000 | 0.966 | 1.000 | 0.010 | 0.454 | 0.605 | -0.009 | 0.441 | 0.795 |
| NAc-shell-rh | 0.004 | 0.404 | 1.000 | 0.005 | 0.633 | 0.751 | 0.003 | 0.753 | 0.847 |
| NAc-core-rh | 0.003 | 0.587 | 1.000 | 0.002 | 0.838 | 0.865 | 0.005 | 0.522 | 0.795 |
| pGP-rh | 0.011 | 0.025 | 0.513 | 0.015 | 0.067 | 0.202 | 0.002 | 0.768 | 0.847 |
| aGP-rh | 0.009 | 0.114 | 0.731 | 0.010 | 0.255 | 0.509 | 0.006 | 0.487 | 0.795 |
| aPUT-rh | 0.005 | 0.057 | 0.569 | 0.011 | 0.053 | 0.202 | -0.001 | 0.862 | 0.890 |
| pPUT-rh | 0.004 | 0.222 | 1.000 | 0.010 | 0.057 | 0.202 | -0.003 | 0.563 | 0.801 |
| aCAU-rh | -0.009 | 0.248 | 1.000 | -0.012 | 0.318 | 0.513 | -0.008 | 0.476 | 0.795 |
| pCAU-rh | -0.024 | 0.010 | 0.318 | -0.013 | 0.335 | 0.513 | -0.025 | 0.080 | 0.426 |
| aHIP-lh | 0.022 | 0.002 | 0.217 | 0.042 | 0.001 | 0.037 | 0.001 | 0.939 | 0.939 |
| pHIP-lh | 0.008 | 0.210 | 1.000 | 0.023 | 0.035 | 0.202 | -0.010 | 0.316 | 0.777 |
| lAMY-lh | 0.013 | 0.058 | 0.569 | 0.011 | 0.337 | 0.513 | 0.012 | 0.297 | 0.777 |
| mAMY-lh | 0.015 | 0.100 | 0.688 | 0.032 | 0.057 | 0.202 | -0.014 | 0.355 | 0.795 |
| THA-DP-lh | 0.012 | 0.095 | 0.688 | 0.012 | 0.284 | 0.513 | 0.009 | 0.423 | 0.795 |
| THA-VP-lh | 0.002 | 0.548 | 1.000 | 0.013 | 0.152 | 0.348 | -0.009 | 0.197 | 0.699 |
| THA-VA-lh | -0.004 | 0.450 | 1.000 | 0.014 | 0.179 | 0.382 | -0.022 | 0.012 | 0.222 |
| THA-DA-lh | 0.001 | 0.866 | 1.000 | 0.025 | 0.069 | 0.202 | -0.027 | 0.024 | 0.255 |
| NAc-shell-lh | 0.002 | 0.630 | 1.000 | 0.003 | 0.817 | 0.865 | 0.004 | 0.720 | 0.847 |
| NAc-core-lh | 0.006 | 0.263 | 1.000 | 0.007 | 0.531 | 0.654 | 0.010 | 0.286 | 0.777 |
| pGP-lh | 0.008 | 0.095 | 0.688 | 0.016 | 0.038 | 0.202 | -0.004 | 0.601 | 0.801 |
| aGP-lh | 0.004 | 0.566 | 1.000 | 0.000 | 0.975 | 0.975 | 0.007 | 0.507 | 0.795 |
| aPUT-lh | 0.008 | 0.029 | 0.513 | 0.007 | 0.405 | 0.581 | 0.010 | 0.098 | 0.450 |
| pPUT-lh | 0.007 | 0.059 | 0.569 | 0.009 | 0.120 | 0.320 | 0.002 | 0.757 | 0.847 |
| aCAU-lh | -0.009 | 0.317 | 1.000 | -0.009 | 0.508 | 0.651 | -0.003 | 0.842 | 0.890 |
| pCAU-rh | -0.016 | 0.164 | 0.983 | -0.006 | 0.759 | 0.865 | -0.017 | 0.373 | 0.795 |
| aHIP-lh | 0.012 | 0.032 | 0.513 | 0.016 | 0.141 | 0.348 | 0.013 | 0.142 | 0.569 |
| pHIP-lh | 0.011 | 0.044 | 0.569 | 0.006 | 0.418 | 0.581 | 0.014 | 0.077 | 0.426 |
| lAMY-lh | 0.006 | 0.076 | 0.659 | 0.026 | 0.004 | 0.059 | -0.015 | 0.070 | 0.426 |
| mAMY-lh | 0.021 | 0.006 | 0.307 | 0.034 | 0.020 | 0.202 | 0.006 | 0.667 | 0.847 |
| THA-DP-lh | 0.008 | 0.216 | 1.000 | 0.021 | 0.061 | 0.202 | -0.005 | 0.583 | 0.801 |
| THA-VP-lh | -0.002 | 0.620 | 1.000 | 0.008 | 0.292 | 0.513 | -0.016 | 0.014 | 0.222 |
| THA-VA-lh | -0.004 | 0.470 | 1.000 | 0.002 | 0.836 | 0.865 | -0.010 | 0.236 | 0.754 |
| THA-DA-lh | 0.000 | 0.966 | 1.000 | 0.010 | 0.454 | 0.605 | -0.009 | 0.441 | 0.795 |
| NAc-shell-lh | 0.004 | 0.404 | 1.000 | 0.005 | 0.633 | 0.751 | 0.003 | 0.753 | 0.847 |
| NAc-core-lh | 0.003 | 0.587 | 1.000 | 0.002 | 0.838 | 0.865 | 0.005 | 0.522 | 0.795 |
| pGP-lh | 0.011 | 0.025 | 0.513 | 0.015 | 0.067 | 0.202 | 0.002 | 0.768 | 0.847 |
| aGP-lh | 0.009 | 0.114 | 0.731 | 0.010 | 0.255 | 0.509 | 0.006 | 0.487 | 0.795 |
| aPUT-lh | 0.005 | 0.057 | 0.569 | 0.011 | 0.053 | 0.202 | -0.001 | 0.862 | 0.890 |
| pPUT-lh | 0.004 | 0.222 | 1.000 | 0.010 | 0.057 | 0.202 | -0.003 | 0.563 | 0.801 |
| aCAU-lh | -0.009 | 0.248 | 1.000 | -0.012 | 0.318 | 0.513 | -0.008 | 0.476 | 0.795 |
| pCAU-lh | -0.024 | 0.010 | 0.318 | -0.013 | 0.335 | 0.513 | -0.025 | 0.080 | 0.426 |

## Supplementary Figure 2. Between group differences in grey matter microstructure adjusting for total intracranial volume

We replicated our main results for between group differences between patients with Lewy body disease (LBD), Parkinson’s with normal cognition (PD-NC) and controls (HC), using General linear models were used with age, sex and total intracranial volume as nuisance covariates and false discovery rate (FDR) correction for multiple comparisons. Colour scale depicts effect size (blue colours decreases in the examined grey matter metric red colours increases in the examined metric). Only statistical significant regions after multiple comparisons correction (FDR-p <0.05) are shown.

**A.** Free water (FWF) changes.

**B.** Tissue-weighted weighted orientation dispersion index changes (tODI). There were no significant changes between PD-NC and HC. There were no significant changes in tNDI between any groups.

**C.** Cortical thickness changes. There were no significant changes between LBD vs PD-NC or PD-NC and HC.

**A**

LBD vs PD-NC

LBD vs HC

PD-NC vs HC

**B**

LBD vs HC

LBD vs PD-NC

LBD vs HC

**C**

## Supplementary Figure 3. Between group differences in grey matter microstructure adjusting for disease duration

To account for the difference in disease duration between LBD and PD-NC participants, we replicated our main results for between group differences between patients with Lewy body disease (LBD), Parkinson’s with normal cognition (PD-NC) and controls (HC), using General linear models were used with age, sex and disease duration as nuisance covariates and false discovery rate (FDR) correction for multiple comparisons. Colour scale depicts effect size (blue colours decreases in the examined grey matter metric red colours increases in the examined metric). Only statistical significant regions after multiple comparisons correction (FDR-p <0.05) are shown.

**A.** Free water (FWF) changes between LBD vs HC and LBD vs PD-NC.

**B.** Tissue-weighted weighted orientation dispersion index changes (tODI) between LBD vs HC and LBD vs PD-NC. There were no significant changes in tNDI between any groups.

**C.** Cortical thickness changes between LBD vs HC and LBD vs PD-NC.

**A**

LBD vs PD-NC

LBD vs HC

**B**

LBD vs PD-NC

LBD vs HC

**C**

LBD vs HC

LBD vs PD-NC

## Supplementary Figure 4. Between group differences in grey matter microstructure including only patients with dementia for LBD comparisons (excluding mild cognitive impairment (MCI).

We replicated our main results for between group differences between only patients with Lewy body dementias (DLB and PDD, excluding those with MCI), Parkinson’s with normal cognition (PD-NC) and controls (HC), using General linear models were used with age and sex as nuisance covariates and false discovery rate (FDR) correction for multiple comparisons. Colour scale depicts effect size (blue colours decreases in the examined grey matter metric red colours increases in the examined metric). Only statistically significant regions after multiple comparisons correction (FDR-p <0.05) are shown.

**A.** Free water (FWF). **B.** Tissue-weighted weighted orientation dispersion index (tODI). There were no significant changes in tNDI. **C.** Cortical thickness.

**A**

LBD vs HC

LBD vs PD-NC

**B**

LBD vs HC

LBD vs PD-NC

**C**

LBD vs HC

## Supplementary Figure 5. Changes in microstructural measures in relation to MMSE and MOCA

Increased cortical free-water fraction (FWF **(A)**) and tissue-weighted orientation dispersion index (tODI **(B)**) were correlated with MMSE (top) and MOCA (bottom) in patients with Lewy body diseases (n=155 including both Parkinson’s disease with normal cognition (PD-NC) and Lewy body dementia (LBD) participants).

General linear models were used with age and sex as nuisance covariates and false discovery rate (FDR) correction for multiple comparisons. Colour scale depicts effect size (blue colours decreases in the examined grey matter metric with increased scores; red colours increases in the examined metric with increased scores). Only statistical significant regions after multiple comparisons correction (FDR-p <0.05) are shown.

**A**

MMSE

MOCA

**B**

MMSE

MOCA

## Supplementary Figure 6. Changes in microstructural measures in relation to disease severity and p-tau217 levels, adjusting for diagnostic group

Increased cortical free-water fraction (FWF) was correlated with cognitive severity metrics in patients with Lewy body diseases (n=155 including both Parkinson’s disease with normal cognition (PD-NC) and Lewy body dementia (LBD) participants), when including diagnostic group (PD-NC, LBD) as covariate. There was no significant association between FWF with total UPRDS score or any motor severity metric (UPDRS part-III and TUG score).

tODI association with cognition did not remain significant after inclusion of diagnostic group as a covariate.

General linear models were used with age, sex and diagnostic group as nuisance covariates and false discovery rate (FDR) correction for multiple comparisons. Colour scale depicts effect size (blue colours decreases in the examined grey matter metric with increased scores; red colours increases in the examined metric with increased scores). Only statistical significant regions after multiple comparisons correction (FDR-p <0.05) are shown.

**A**

Combined cognitive score

MMSE

MOCA

p-tau217

## Supplementary Figure 7. Changes in microstructural measures in relation to disease severity and p-tau217 levels, adjusting for diagnostic group and disease duration

Increased cortical free-water fraction (FWF) was correlated with cognitive severity metrics in patients with Lewy body diseases (n=155 including both Parkinson’s disease with normal cognition (PD-NC) and Lewy body dementia (LBD) participants), when including diagnostic group (PD-NC, LBD) and disease duration as covariates. There was no significant association between FWF with any motor severity metric (UPDRS part-III and TUG score).

General linear models were used with age, sex, diagnostic group and disease duration as nuisance covariates and false discovery rate (FDR) correction for multiple comparisons. Colour scale depicts effect size (blue colours decreases in the examined grey matter metric with increased scores; red colours increases in the examined metric with increased scores). Only statistically significant regions after multiple comparisons correction (FDR-p <0.05) are shown.

Combined cognitive score

MMSE

UPDRS total

MOCA

p-tau217

## Supplementary Figure 8. Longitudinal changes in Free-water fraction between PD-poor outcomes and controls

Greater increases in cortical free-water fraction (FWF) were seen longitudinally in patients with Parkinson’s disease with normal cognition at baseline, who developed cognitive impairment (PD-poor outcomes) during 3 year follow up compared to controls.

General linear models were used to assess longitudinal differences in group * time interactions between patients with Parkinson’s disease that developed mild cognitive impairment or dementia (PD poor outcomes, n=31) compared to controls (n=27); age and sex as nuisance covariates, false discovery rate (FDR) correcting for multiple comparisons.

Only statistically significant regions after multiple comparisons correction (FDR-p <0.05) are shown.

## Supplementary Figure 9. Longitudinal changes in Free-water fraction in relation to MOCA

Rate of change in cortical free-water fraction (FWF) was correlated with rate of changes in combined cognitive score and also with rate of changes in MOCA in patients with Parkinson’s disease with normal cognition at baseline, followed up over 3 years, n=100). There were no significant correlations with MMSE (FDR-p>0.05).

General linear models were used with age and sex as nuisance covariates and false discovery rate (FDR) correction for multiple comparisons. Only statistically significant regions after multiple comparisons correction (FDR-p <0.05) are shown.

## Supplementary Table 5. Parameter estimation for Sample size calculations

Sample size requirements for a hypothetical disease modifying trial were calculated for each imaging metric using simulation-based power calculations of subject-level longitudinal slopes equivalent to testing the treatment*time interaction under a linear mixed-effects model. Cognitive outcomes were modelled as linear change over time measured at baseline, 18- and 36-months. Between-subject variability and within-subject residual variance components were derived empirically from our longitudinal mixed models for the cortical region showing the maximum effect size (partial r). Simulated datasets reflected a planned trial design with assessments at baseline, 18- and 36-months similar to our longitudinal cohort. Treatment effects were specified as proportional reductions in the excess rate of cognitive decline between PD poor and PD good outcomes, reflecting treatment-induced differences in longitudinal slope. Subject-specific linear slopes were estimated from simulated longitudinal data and compared between treatment and control groups using two-sample tests. For each candidate sample size, 500 simulated trials were generated, with refitting of mixed effects model. The minimum sample size per arm achieving 80% power with statistical significance α=0.05 is reported.

**Combined cognitive scores changes in PD poor vs PD good outcomes**

| **Session** | **PD poor outcomes**  **(n= 31)** | **PD good outcomes**  **(n=69)** |
| --- | --- | --- |
| **Session 1** | -0.917 (0.852) | -0.001 (0.603) |
| **Session 2** | -0.341 (0.702) | -0.458 (1.109) |
| **Session 3** | -1.108 (1.048) | -0.085 (0.568) |

These cognitive scores were used to calculate slopes of decline in PD poor vs good outcomes:

- PD Poor outcomes: -0.064
- PD Good outcomes: 0.029
- Difference slope (PD poor vs good outcomes): -0.092

This means that natural decline in progressors is -0.092 units/year relative to patients with stable cognition.

**Free water fraction (FWF)**

The region with the maximum effect size (partial r=0.257, false discovery rate (FDR)-corrected p-value, q=0.009) was used to calculate sample sizes for a hypothetical clinical trial. This corresponded to parcel 4, 7Networks_LH_Vis_4, MNI coordinates -10, -68, -4, left visual association Broadman area 19. Residual SD (0.036) and random intercept SD (0.037) from a mixed linear model corrected for age and sex and random subject-specific intercept were used to simulate longitudinal data.

**Cortical thickness**

There were no statistically significant longitudinal differences between PD good vs PD poor outcomes in cortical thickness following FDR correction. Therefore, for the calculation of sample sizes required for clinical trials we used the region with the maximum effect size partial r=-0.155 (p-value q=0.505, uncorrected p-value p=0.018; parcel 7Networks_Lh_Default_PFC_11, MNI coordinates -40, 18, 50, left frontal eye field). Residual SD (0.068) and random intercept SD (0.105) from a mixed linear model corrected for age and sex and random subject-specific intercept were used to simulate longitudinal data.

**Tissue-weighted orientation dispersion index (tODI)**

There were no statistically significant differences between PD good vs PD poor outcomes in tODI following FDR correction with bidirectional changes seen in uncorrected results. To calculate sample sizes for clinical trials we used the region with maximum absolute effect size partial r=-0.187, q=0.667, p=0.004; parcel 7Networks_RH_Default_PFCdPFCm_5, MNI coordinates 16, 46, 44, right dorsolateral prefrontal cortex Broadmann area 9. Residual SD (0.016) and random intercept SD (0.045) from a mixed linear model corrected for age and sex and random subject-specific intercept were used to simulate longitudinal data.

## URLs:

AMICO - <https://github.com/daducci/AMICO>

Brain Stat: <https://brainstat.readthedocs.io/en/master/>

MRtrix: <https://www.mrtrix.org/>

MRIQC: https://mriqc.readthedocs.io/

Niftyreg: <https://github.com/KCL-BMEIS/niftyreg>

Tissue-weighted means: <https://github.com/tdveale/TissueWeightedMean>

## References

1.	Creavin ST, Wisniewski S, Noel‐Storr AH, et al. Mini‐Mental State Examination (MMSE) for the detection of dementia in clinically unevaluated people aged 65 and over in community and primary care populations [Internet]. Cochrane Database of Systematic Reviews 2016;(1)[cited 2019 Feb 15 ] Available from: https://www.cochranelibrary.com/cdsr/doi/10.1002/14651858.CD011145.pub2/abstract

2.	Dalrymple-Alford JC, MacAskill MR, Nakas CT, et al. The MoCA: well-suited screen for cognitive impairment in Parkinson disease. Neurology 2010;75(19):1717–1725.

3.	Stroop JR. Studies of interference in serial verbal reactions. Journal of Experimental Psychology 1935;18(6):643–662.

4.	Wechsler, D. Wechsler Adult Intelligence Scale-Fourth Edition [Internet]. Fourth. San Antonio, TX: NCS Pearson; 2008.[cited 2019 Feb 6 ] Available from: https://www.pearsonclinical.com/psychology/products/100000392/wechsler-adult-intelligence-scalefourth-edition-wais-iv.html

5.	Rende B, Ramsberger G, Miyake A. Commonalities and differences in the working memory components underlying letter and category fluency tasks: a dual-task investigation. Neuropsychology 2002;16(3):309–321.

6.	Warrington EK. The Graded Naming Test: A Restandardisation. Neuropsychological Rehabilitation 1997;7(2):143–146.

7.	Hooper H. Hooper Visual Organization Test (VOT) Manual. Los Angeles: CA: Western Psychological Services; 1983.

8.	Benton AL, Varney NR, Hamsher K deS. Visuospatial Judgment: A Clinical Test. Arch Neurol 1978;35(6):364–367.

9.	Hannaway N, Zarkali A, Leyland L-A, et al. Visual dysfunction is a better predictor than retinal thickness for dementia in Parkinson’s disease [Internet]. J Neurol Neurosurg Psychiatry 2023;[cited 2023 July 14 ] Available from: https://jnnp.bmj.com/content/early/2023/04/20/jnnp-2023-331083

10.	Walker MP, Ayre GA, Cummings JL, et al. The Clinician Assessment of Fluctuation and the One Day Fluctuation Assessment Scale. Two methods to assess fluctuating confusion in dementia. Br J Psychiatry 2000;177:252–256.

11.	Pfeffer RI, Kurosaki TT, Harrah CH, et al. Measurement of functional activities in older adults in the community. J Gerontol 1982;37(3):323–329.

12.	Stiasny-Kolster K, Mayer G, Schäfer S, et al. The REM sleep behavior disorder screening questionnaire--a new diagnostic instrument. Mov. Disord. 2007;22(16):2386–2393.

13.	Papapetropoulos S, Katzen H, Schrag A, et al. A questionnaire-based (UM-PDHQ) study of hallucinations in Parkinson’s disease. BMC Neurology 2008;8:21.

14.	Tomlinson CL, Stowe R, Patel S, et al. Systematic review of levodopa dose equivalency reporting in Parkinson’s disease. Movement Disorders 2010;25(15):2649–2653.

15.	Emre M, Aarsland D, Brown R, et al. Clinical diagnostic criteria for dementia associated with Parkinson’s disease. Mov. Disord. 2007;22(12):1689–1707; quiz 1837.

16.	Postuma RB, Berg D, Stern M, et al. MDS clinical diagnostic criteria for Parkinson’s disease. Movement Disorders 2015;30(12):1591–1601.

17.	Goetz CG, Tilley BC, Shaftman SR, et al. Movement Disorder Society-sponsored revision of the Unified Parkinson’s Disease Rating Scale (MDS-UPDRS): scale presentation and clinimetric testing results. Mov. Disord. 2008;23(15):2129–2170.

18.	Creavin ST, Wisniewski S, Noel-Storr AH, et al. Mini-Mental State Examination (MMSE) for the detection of dementia in clinically unevaluated people aged 65 and over in community and primary care populations. Cochrane Database Syst Rev 2016;2016(1):CD011145.

19.	Zarkali A, Adams RA, Psarras S, et al. Increased weighting on prior knowledge in Lewy body-associated visual hallucinations [Internet]. Brain Commun 2019;1(1)[cited 2019 Oct 29 ] Available from: https://academic.oup.com/braincomms/article/1/1/fcz007/5532510

20.	Hannaway N, Zarkali A, Bhome R, et al. Neuroimaging and plasma biomarker differences and commonalities in Lewy body dementia subtypes [Internet]. 2025;2025.01.24.634687.[cited 2025 May 15 ] Available from: https://www.biorxiv.org/content/10.1101/2025.01.24.634687v1
